# Academic Achievement in Children with ADHD: the Role of Processing Speed and Working Memory

**DOI:** 10.1007/s10802-025-01346-6

**Published:** 2025-07-15

**Authors:** An-Katrien Hulsbosch, Saskia Van der Oord, Gail Tripp

**Affiliations:** 1 Human Developmental Neurobiology Unit, Okinawa Institute of Science and Technolology (OIST) Graduate University, Onna, Okinawa Japan; 2 Behavior, Health and Psychopathology, KU Leuven, Leuven, Belgium; 3 Child and Youth Institute, KU Leuven, Leuven, Belgium

**Keywords:** ADHD, Academic achievement, Processing speed, Working memory

## Abstract

Academic underachievement is often reported in children with attention-deficit/hyperactivity disorder (ADHD). Research shows working memory (WM), and processing speed (PS) have a predictive role in their academic difficulties. Recent evidence suggests both cognitive functions are related, where slower PS underlies WM deficits. This relationship is not accounted for in the current literature on academic underachievement in children with ADHD. In the current study, the role of PS and WM in the association between ADHD symptom severity and academic achievement is investigated in a sample of 504, 6 to 12 years old children diagnosed with ADHD. Academic achievement is assessed across three subjects (mathematics, reading and spelling), by three measurement methods (standardized tests, parent, and teacher ratings). In addition, the role of PS is investigated in the relationship between ADHD symptom severity and WM. Findings show the association between inattention symptom severity and achievement on all three academic subjects is statistically mediated by PS and WM sequentially. For mathematics and spelling performance, PS was a single mediator in this association. Further, PS statistically mediated the relation between inattention symptom severity and WM performance. Hyperactivity/impulsivity symptom severity predicted mathematics performance directly, but none of the indirect effects were significant. The current results show that PS plays an important role in the academic achievement of children with increased inattention symptoms, as well as the WM deficits often reported in this population. These findings have important implications for theoretical accounts of ADHD, as well as academic interventions, currently focusing primarily on WM deficits.

**Supplementary Information:**

The online version contains supplementary material available at 10.1007/s10802-025-01346-6.

## Introduction

Attention Deficit/Hyperactivity Disorder (ADHD) is a common neurodevelopmental condition with a prevalence rate estimated around 5.9% in children and 2.5% in adults (Faraone et al., 2021). It is characterized by developmentally inappropriate levels of inattention, hyperactivity and impulsivity that cause significant impairments in everyday functioning (American Psychiatric Association, 2022). High comorbidity rates of autism spectrum disorder (ASD), learning disorders, oppositional defiant disorder (ODD) and conduct disorder (CD) are reported amongst others (Gnanavel et al., 2019).

Academic underachievement is widely reported in samples of children, adolescents, and young adults with ADHD (Frazier et al., 2007; Garner et al., 2013). Individuals with ADHD show impairments across academic subjects, such as reading, mathematics, and spelling, measured through standardized tests, and parent and teacher ratings (Frazier et al., 2007; Garner et al., 2013). These academic impairments are continuously reported throughout development, showing a decline in performance with increasing age (Murray et al., 2017) and the greatest impairments reported in those not receiving treatment (Arnold et al., 2020). Individuals with ADHD are more likely to receive special educational support, show lower academic attainment and increased drop-out rates of school, and miss more classes in high school (Barbaresi et al., 2007; Fleming et al., 2017; Kent et al., 2011).

To better understand and manage the academic underachievement of children with ADHD, it is important to investigate predicting factors. Research indicates several cognitive functions are related to academic achievement, both in neurotypical children (Alloway & Alloway, 2010; Caemmerer et al., 2018), and children with ADHD (Braaten et al., 2020; Simone et al., 2018). One such cognitive function is working memory (WM), which is the ability to concurrently process and store information. A distinction is often made between verbal WM and visual-spatial WM, in which information is processed through a phonological loop or visuospatial sketchpad respectively, with each potentially contributing to different academic outcomes (Sowerby et al., 2011).

Research shows a combined verbal and visual-spatial WM index is predictive of reading, mathematics, and writing performance (Caemmerer et al., 2018). Also longitudinally, verbal WM performance is predictive of both reading and mathematics performance, explaining significant variance over and above IQ (Alloway & Alloway, 2010). Verbal and visual-spatial WM deficits are well established in children with ADHD (Sowerby et al., 2011). Both verbal and visual-spatial WM ability have been shown to be significantly related to academic achievement in children with ADHD, with ratings of inattention and/or hyperactive/impulsive symptoms showing no relation to academic skills when WM is included as predictor (Simone et al., 2018). In another study, verbal and visual-spatial WM mediated the relationship between ADHD diagnosis and ratings of academic achievement (Sjöwall & Thorell, 2014), providing further evidence for the role of WM in academic achievement in this group.

Another cognitive function often considered in relation to academic achievement, is processing speed (PS), which is the ability to quickly and accurately perform simple mental operations. A study by Geary (2011) showed PS is significantly related to mathematics and reading performance, which remained significant when controlling for intelligence and WM. Moreover, research has shown PS is a significant predictor of school grades and makes a unique contribution to academic achievement beyond intelligence (Dodonova & Dodonov, 2012). Deficits in PS are also found to be related to ADHD symptoms, although mostly reported in children with inattentive symptoms (Kibby et al., 2019) or the inattentive presentation ADHD (Goth-Owens et al., 2010). A recent meta-analysis showed that poorer PS in ADHD is related to poorer academic achievement, confirming the potential link between them (Cook et al., 2018).

However, a relation between PS and WM is proposed, which is typically not considered when investigating the effect of those cognitive abilities on academic achievement in children with ADHD. More precisely, the time-based resource-sharing (TBRS) model (Portrat et al., 2009) hypothesizes that slower PS leads to a higher cognitive load, as cognitive capacities are being occupied by processing of the task. This in turn prevents refreshing of memory content, consequently impairing WM performance (Portrat et al., 2009). This model may be of importance for children with ADHD, and research suggests that in these children, slower PS may be a mediator of poorer WM performance (Karalunas & Huang-Pollock, 2013; Weigard & Huang-Pollock, 2017). Karalunas and Huang-Pollock (2013) reported that PS, measured through an estimate of information processing efficiency, partially mediated the relation between ADHD diagnosis and WM capacity. This hypothesis is also supported by an experimental study that found an effect of PS on cognitive load, thereby impacting WM recall in children with ADHD (Weigard & Huang-Pollock, 2017).

Considering the potential mediating effect of both PS and WM in relation to academic achievement, one study found that the association between premature birth and lower math and reading achievement was fully mediated through slower PS and poorer WM sequentially (verbal and visual-spatial WM combined). This result suggests a cascade of effects in this population, with slower PS contributing to poorer WM performance (Rose et al., 2011). Another study found an elevated risk for slow PS in children with ADHD, and found a direct effect of PS on academic achievement, as well as a significant indirect effect through verbal WM, indicating partial mediation (Braaten et al., 2020). This finding was replicated in a more recent study, showing both verbal and visual-spatial WM to be partial mediators in the relation between cognitive PS and math fluency in children with ADHD (Lee, 2024). Although both studies showed partial mediation of WM in the relation between PS and academic achievement, none of these studies investigated the effects of ADHD symptoms as predictor in this relation. Furthermore, investigated samples were small, or only one academic subject was considered when investigating academic achievement.

Another limitation of the existing research investigating academic achievement in ADHD, is the inclusion of only one measurement method to assess academic achievement. Either standardized tests are included (e.g., Braaten et al., 2020; Lee, 2024) or ratings made by parents/teachers comparing the child with their peers (Sjöwall & Thorell, 2014). Moreover, even when multiple measures of academic achievement are collected, analyses are carried out separately (Simone et al., 2018). The integration of multiple measurement methods is increasingly acknowledged as providing a more complete assessment compared to the use of one measurement method only (Moon, 2019). This method, called *triangulation*, is shown to increase validity and reliability of research findings as it provides a more comprehensive picture of the assessed concept (Moon, 2019). To date, it has not been implemented in studies addressing predictors of academic underachievement in ADHD.

The current study investigated the role of PS and WM separately, as well as the sequential role of both, in the relationship between ADHD symptom severity and academic achievement. The role of PS in the relationship between ADHD symptom severity and WM was investigated as well. Measures of multiple academic subjects (i.e., reading, mathematics, and spelling) were included across multiple measurement methods. The measurement model of academic achievement was investigated in advance of the mediation model using the triangulation method (Moon, 2019), to examine the validity and reliability before implementation. Convergent and discriminant validity of the different academic subjects, as well as measurement method effects were investigated. The WM index from the Wechsler Intelligence Scale for Children, edition IV (WISC-IV; Wechsler, 2003) or edition V (WISC-V; Wechsler, 2014) were used to assess WM performance, but supplementary analyses were conducted for verbal and visual-spatial WM separately for those completing edition V. Lastly, although mostly symptoms of inattention have been related to several of the included cognitive functions and academic achievement, both inattention symptom severity and hyperactivity/impulsivity symptom severity were investigated separately, to assess potential differential effects of both symptom domains. We expect PS to be a (partial) statistical mediator in the relation between ADHD symptom severity and academic achievement. We also expect WM to be a (partial) statistical mediator in this relationship. Moreover, we expect the serial mediation effect, through both PS and WM sequentially, to be statistically significant. Lastly, we expect the relationship between ADHD symptom severity and WM to be (partially) statistically mediated by PS.

## Methodology

Ethical approval for the study was obtained from the Human Subjects Research Review Committee at the Okinawa Institute of Science and Technology (OIST) Graduate University, Japan. Parents provided written consent, and children gave written assent to participate.

### Participants and Procedure

Data for the present study was collected through the ADHD Research Center of the Okinawa Institute of Science and Technology (OIST) Graduate University, between 2010 and 2024. A total of 504 English speaking children (339 male, 165 female), aged 6 to 12 years old, were included in the current sample (Table 1). Children took part in a variety of studies, but all completed the necessary measures of academic performance and cognitive functioning, and parent and teacher ratings were collected for all participants. For some of the standardized tests or questionnaires, different versions were administered throughout the data collection period as new and updated version became available over time. Children were primarily US citizens temporarily residing in Japan, and all showed sufficient levels of English to participate in the present study. All children were volunteers recruited for research participation. Parents of participating children became aware of the research through a variety of sources, including teachers and in some cases pediatricians or psychiatrists.

**Table 1:** Detailed description of the sample on demographic characteristics, clinical characteristics and descriptive values of included measures

	*N* = 504
Age (years), M (*SD*)	8.73 (1.73)
Male sex, *n *(%)	339 (67.3)
Race, *n *(%)	*Missing n = *137
Caucasian	207 (56.40)
Native Hawaiian/other Pacific	3 (0.80)
Black/African American	22 (6.00)
Hispanic/Latino	22 (6.00)
Japanese	6 (1.60)
Asian Other	4 (1.10)
Mixed	103 (28.10)
Education Language, *n *(%)	*Missing n = *143
English	351 (97.23)
Japanese	10 (2.77)
Medication, *n *(%)	
Psychostimulant	114 (22.62)
Non-psychostimulant	7 (1.39)
Comorbidity, *n *(%)	
ODD	25 (4.96)
Tic disorder	1 (0.20)
Learning disorder	41 (8.13)
Autism spectrum disorder	24 (4.76)
Anxiety/Mood disorder	53 (10.52)
ADHD symptom sum, M (*SD*)	
Inattentive	17.45 (5.32)
Hyperactive/Impulsive	13.47 (6.50)
Cognitive functioning, M (*SD*)	
Processing Speed Index	97.10 (14.96)
Working Memory Index	96.40 (13.07)
Digit Span Scaled Score (*WISC-5)*	9.10 (2.50)
Picture Span Scaled Score (*WISC-5)*	10.08 (3.03)
Academic functioning, M (*SD*)	
Standardized Test – Reading	101.74 (15.80)
Standardized Test – Mathematics	100.07 (13.21)
Standardized Test – Spelling	97.77 (14.05)
Parent Rating – Reading	2.75 (0.86)
Parent Rating – Mathematics	2.77 (0.82)
Teacher Rating – Reading	2.65 (1.17)
Teacher Rating – Mathematics	2.63 (0.97)
Teacher Rating - Spelling	2.30 (0.99)

All participants had a full scale IQ of at least 70, measured with the WISC-IV (Wechsler, 2003) or WISC-V (Wechsler, 2014). Children met DSM-IV or DSM-V diagnostic criteria for ADHD, established through multimethod, multi-informant diagnostic assessments. Diagnosis was based on a clinical and semi-structured diagnostic interview (Schedule for Affective Disorders and Schizophrenia for School-Age Children – Present and Lifetime Version [K-SADS]; Kaufman et al., 1997, 2016), parent and teacher ratings of ADHD symptoms (Swanson, Nolan & Pelham ADHD rating scale [SNAP], Swanson et al., 2012; or Conners Behavioral Rating Scale [CBRS], Conners, 2008) and behavioral observations of the child during the assessment. Children were required to display at least six symptoms of inattention and/or hyperactivity/impulsivity in at least one setting and evidence of symptoms in a second setting (i.e., home, school, or clinic), causing significant impairment. Final diagnostic decisions were made by a US licensed clinical psychologist based on all available data. There was no exclusion of children based on comorbid conditions. Children with ADHD taking stimulant medication discontinued medication use at least 24 h prior to testing to allow for wash-out (Greenhill et al., 2002).

### ADHD Symptom Severity

ADHD symptom severity scores were measured by either the Swanson, Nolan & Pelham ADHD Rating Scale (SNAP; Swanson et al., 2012; *n* = 157) or the Conners Behavioral Rating Scale (Conners, 2008; *n* = 347). Both questionnaires included the same DSM-IV/V items and used a similar 4-point rating scale. Inattention and hyperactivity/impulsivity symptom severity scores were calculated as the sum of the raw parent-ratings on the DSM-IV/V inattention and hyperactivity/impulsivity symptom items from these scales.

### Processing Speed

The Processing Speed Index (PSI) of the WISC-IV (*n* = 134) or WISC-V (*n* = 369) was used to measure processing speed. For both versions, the PSI was calculated based on the norm score of two subtests, Coding and Symbol Search. The WISC-IV/V is a standardized measure for intellectual functioning in children (aged 6 to 16), and age standardized norm scores for the PSI were used for all test versions (Wechsler, 2003, 2014).

### Working Memory

The Working Memory Index (WMI) of the WISC-IV (*n* = 134) or WISC-V (*n* = 369) was used to measure working memory performance. The WMI was calculated based on the norm score of two subtests: Digit Span and Letter-Number Sequencing for WISC-IV, Digit Span and Picture Span for WISC-V. To assess the differential effect of verbal WM and visual-spatial WM, the norm scores of the Digit Span (*verbal*) and Picture Span (*Visual-Spatial*) subtests of the WISC-V were used (see Supplementary Results).

### Academic Achievement

#### Standardized Tests

Academic achievement in reading, mathematics and spelling was measured with the Wide Range Achievement test 4 (WRAT-4; Wilkinson & Robertson, 2006; *n* = 129), or the Wechsler Individual Achievement test edition 3 (WIAT-3; Wechsler, 2009; *n* = 148) or edition 4 (WIAT-4; Wechsler, 2020; *n* = 209). Reading performance was assessed via the Word Reading subtest for the WRAT-4, WIAT-3, or WIAT-4. Mathematics performance was assessed via the Math Computation (WRAT-4) or the Numerical Operations subtest (WIAT-3 and WIAT-4). Spelling performance was assessed via the Spelling subtest for WRAT-4, WIAT-3, or WIAT-4. Grade standardized norm scores were used for all test versions and editions.

#### Parent Ratings

Academic achievement in reading and mathematics was measured through parent ratings, where parents rated their children’s performance on a 4-point rating scale for both academic subjects (1 = “failing”, 2 = “below average”, 3 = “average”, 4 = “above average”). Raw scores were used as outcome measure. Parent ratings for spelling performance were not collected.

#### Teacher Ratings

Academic achievement in reading, mathematics and spelling was measured through teacher ratings, where teachers rated the child’s performance for each academic subject on a 5-point rating scale (1 = “far below grade”, 2 = “somewhat below grade”, 3 = “at grade level”, 4 = “somewhat above grade”, 5 = “far above grade”). Raw scores were used as outcome measure.

### Analytical Strategy

Statistical analyses were performed using R (R Core Team, 2024). Descriptive analyses were conducted on the included variables to investigate the distribution of the data. For several of the variables used in the statistical models, different versions of standardized tests or questionnaires were administered during the data collection period (i.e., ADHD symptom severity scores, standardized tests for academic achievement, processing speed and working memory). Measurement invariance across these different versions was tested for the appropriate models based on the variables included, using multigroup Structural Equation Modeling (SEM). All levels of measurement invariance were tested (i.e., configural, metrical, scalar, and strict), metrical invariance was minimally required for the current analyses, assuring factor loadings could be equalized across different test/questionnaire versions. For the structural models, it was additionally investigated if direct and indirect pathways could be equalized across the multiple test versions (see Supplement A for detailed information).

**Fig. 1:**
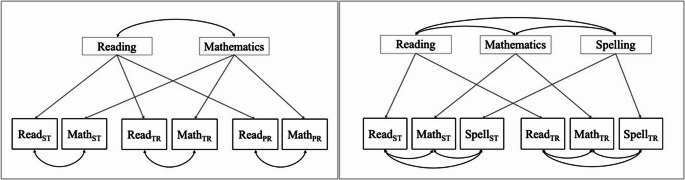
Visual representation of measurement model 1 (left) and measurement model 2 (right) *Note.* ST = standardized test, TR = teacher rating, PR = parent rating

First, two measurement models were fitted ahead of the structural mediation model, to investigate the validity of the used measurement methods for academic achievement (see Fig. 1). Academic performance was examined for three academic subjects (i.e., reading, mathematics, and spelling). Three measurement methods were used for each academic subject (i.e., standardized testing, parent rating, and teacher rating), except parent ratings for spelling were not available. To investigate convergent and discriminant validity of the different academic subjects, two multitrait-multimethod (MTMM) models were tested. Measurement model 1 included two academic subjects (i.e., reading and mathematics) across all three measurement methods. Measurement model 2 included all three academic subjects, but only two measurement methods (i.e., standardized test and teacher rating), as parent ratings for spelling were unavailable. In measurement model 2, factor loadings within the same academic subject were constrained to be equal to guarantee model specification and identification (Kyriazos, 2018b). The two measurement models were tested using the correlated uniqueness approach, in which measurement method effects are accounted for as correlations between the error terms of items from the same method. This method was chosen in favor of the correlated methods approach (i.e., where method factors are specified to investigate method effects), as it allows for a smaller number of measurement methods to be included, and less often results in improper solutions (Brown, 2006; Kyriazos, 2018b).

**Fig. 2:**
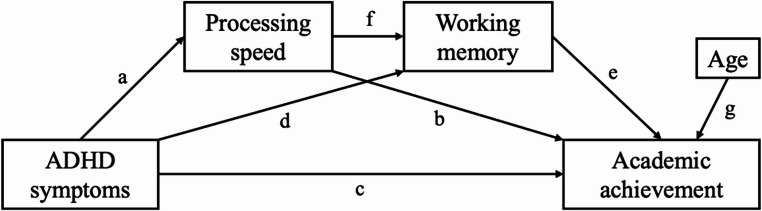
Visual representation of the serial mediation model investigated

Second, different Structural Equation Models (SEM) were tested to investigate the role of PS and WM in the relation between ADHD symptom severity and academic performance (see Fig. 2). Academic performance was operationalized depending on the outcome of the two measurement models. A serial mediation model was used, where ADHD symptom severity scores were entered as the predictor, and PS and WM as the mediators. Based on previous research on the relation between both cognitive functions, PS was entered first and WM second (Braaten et al., 2020; Weigard & Huang-Pollock, 2017, see Fig. 2). One direct and three indirect pathways were estimated for the different outcome variables of academic performance. Pathway *c* represents the direct pathway with ADHD symptom severity as predictor of academic performance. Two simple indirect mediation pathways were included, with the relation between ADHD symptom severity and academic performance mediated through PS (a*b) or through WM (d*e). Next, a serial mediation pathway was included, where the relation between ADHD symptom severity and academic performance is mediated through both PS and WM (a*f*e). Lastly, to investigate the mediating role of PS in the relation between ADHD symptom severity and WM, an additional indirect pathway (a*f) was investigated. Age was inserted as a control variable, and a general pathway from age to academic performance (*g*) was inserted. Full mediation occurred when the indirect pathway was significant, but the direct pathway was not. Partial mediation occurred when both the indirect and direct pathways were significant (Preacher & Hayes, 2008). Bootstrap procedure (5000 draws) was used to evaluate the significance of the indirect pathways. Indirect pathways were significant when their 95% confidence interval (CI) did not contain the value zero. When multiple subjects of academic performance are included simultaneously in the structural model, there will be multiple pathways from the predictor (*c*) and mediators (*b*,* e*) to the academic performance subjects, allowing investigation of direct and indirect pathways specifically for each academic subject. Each structural model will be fitted twice, for symptoms of inattention and hyperactivity/impulsivity separately. Model invariance was investigated across parent-reported sex, assessing whether direct and indirect pathways could be equalized across male and female participants, using the same method as described above. In addition, the models will be fitted separately for the indicators of verbal WM and visual-spatial WM for the subsample of participants (*n* = 369) who completed the WISC-V as it includes subtests for both (the WISC-IV WM index compromises two verbal WM subtests; see Supplement B).

For all models investigated, model fit was considered good based on the following indices and criteria: (1) a non-significant chi-square test or χ²/df < 3, (2) a Comparative Fit Index (CFI) close to 0.95 or greater, (3) a Root Mean Square Error Approximation (RMSEA) close to 0.06 or below, and (4) a Standardized Mean Square Residual (SMSR) close to 0.08 or below (Brown, 2006). When necessary, Modification Indices (MI) will be used to investigate specific locations of model misfit, with a chi-square value of 3.84 or greater suggesting certain parameters to be freed to significantly improve model fit. Full Information Maximum Likelihood (FIML) will be used to handle missing data, as missing data was considered Missing at Random (MAR). The estimation method used was Maximum Likelihood (ML). All observed variables were standardized before entering the analysis, as measurement scales varied substantially across the different variables. The correlation matrix for the different variables as included in the models can be found in Supplement C. Each latent variable was determined by fixing its variance to 1. Standardized estimates of the various parameters were obtained and used for interpreting the results.

## Results

### Measurement Invariance

Measurement invariance across multiple versions of the different tests/questionnaires used was investigated and results showed appropriate levels of invariance across all models and measurements used. For each model, at least metric invariance was found, assuring factor loadings could be equalized across the different versions administered. Multigroup SEM also showed direct and indirect effects could be equalized across test/questionnaire versions for all serial mediation models. Therefore, different versions of tests/questionnaires were taken together into one analysis (see Supplement A).

### Measurement Model Academic Performance

Fit indices of the first (i.e., 2 academic subjects, 3 methods) and second (i.e., 3 academic subjects, 2 methods) measurement model indicated excellent model fit (see Table 2). Convergent validity of the different measurement methods for the academic subjects was confirmed, as all factor loadings were substantial and significant for both models (see Tables 3 and 4). The magnitude of the method effects ranged from small to medium across the models, operationalized as correlated uniqueness between indicators of the same measurement method (see Tables 3 and 4). All method effects were smaller compared to the factor loadings, indicating convergent validity holds when adjusting for method effects (see Tables 3 and 4). The moderate correlations between mathematics and both reading (measurement model 1: *r* =.61, *p* <.001; measurement model 2: *r* =.62, *p* <.001) and spelling (measurement model 2: *r* =.63, *p* <.001), indicated moderate discriminant validity between these academic subjects. Poor discriminant validity was found between the latent factors of reading and spelling in the second measurement model, as the correlation between both was very strong (*r* =.93, *p* <.001). However, model fit of a two-factor model, where the factors of reading and spelling are collapsed into one factor consisting out of four measures, was significantly worse compared to the original three-factor model (see Table 5). Moreover, modification indices for this model showed that model fit would improve significantly when the two measures of reading, as well as the two measures of spelling, were allowed to have additional shared variance on top of the general factor. This indicates that the two measures of both reading and spelling share variance not accounted for by the general factor, further justifying the implementation of the three-factor model.

**Table 2:** Fit indices for the two MTMM models as shown in Fig. 1

Model fit index	MTMM model 1	MTMM model 2
χ² (df)	4.85(5)	5.87(3)
CFI	1.00	0.99
RMSEA	0.00	0.04
SMSR	0.02	0.02

*p <.05, **p <.01, ***p <.001

**Table 3:** Standardized factor loadings and correlated uniqueness between indices of the same measurement method for MTMM model 1

	Factor loadings		Correlated uniqueness		
	Reading	Math	M_ST_	M_PR_	M_TR_
R_ST_	0.71***		0.10		
R_PR_	0.79***			0.14*	
R_TR_	0.86***				0.43***
M_ST_		0.65***			
M_PR_		0.70***			
M_TR_		0.73***			

R = reading; M = mathematics; ST = standardized test; PR = parent rating; TR = teacher rating

*p <.05, **p <.01, ***p <.001

**Table 4:** Standardized factor loadings and correlated uniqueness between indices of the same measurement method for MTMM model 2

	Factor loadings			Correlated Uniqueness			
	Reading	Math	Spelling	M_ST_	S_ST_	M_TR_	S_TR_
R_ST_	0.83***			−0.07	0.52***		
R_TR_	0.73***					0.50***	0.40***
M_ST_		0.66***			0.14*		
M_TR_		0.67***					0.33**
S_ST_			0.74***				
S_TR_			0.73***				

R = reading; M = mathematics; S = spelling; ST = standardized test; TR = teacher rating

*p <.05, **p <.01, ***p <.001

**Table 5:** Comparison of the three -actor (i.e., mathematics, reading, spelling) and two-factor (i.e., mathematics and language) measurement models

Model	χ² (df)	CFI	RMSEA	SMSR	Δχ²	Δdf	*p*-value
Three factors	5.87 (3)	0.99	0.04	0.02			
Two factors	15.57 (5)	0.99	0.07	0.03	9.70	2	0.009**

*p <.05, **p <.01, ***p <.001

### Serial Mediation Analysis

**Fig. 3:**
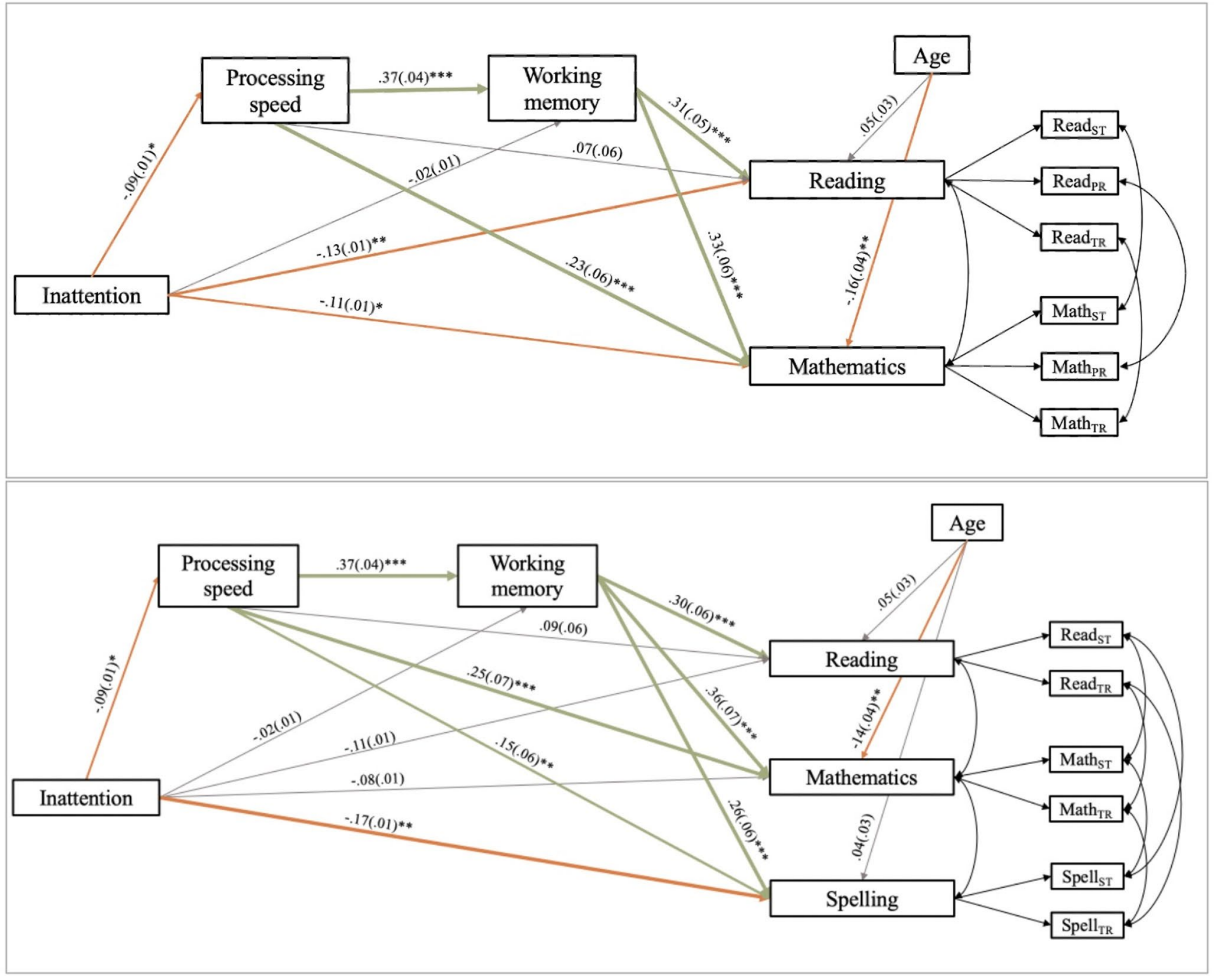
Visual representation of serial mediation model 1 (top) and serial mediation model 2 (bottom) for inattention symptom severity as predictor. *Note. *ST = standardized test, TR = teacher rating, PR = parent rating

**Fig. 4:**
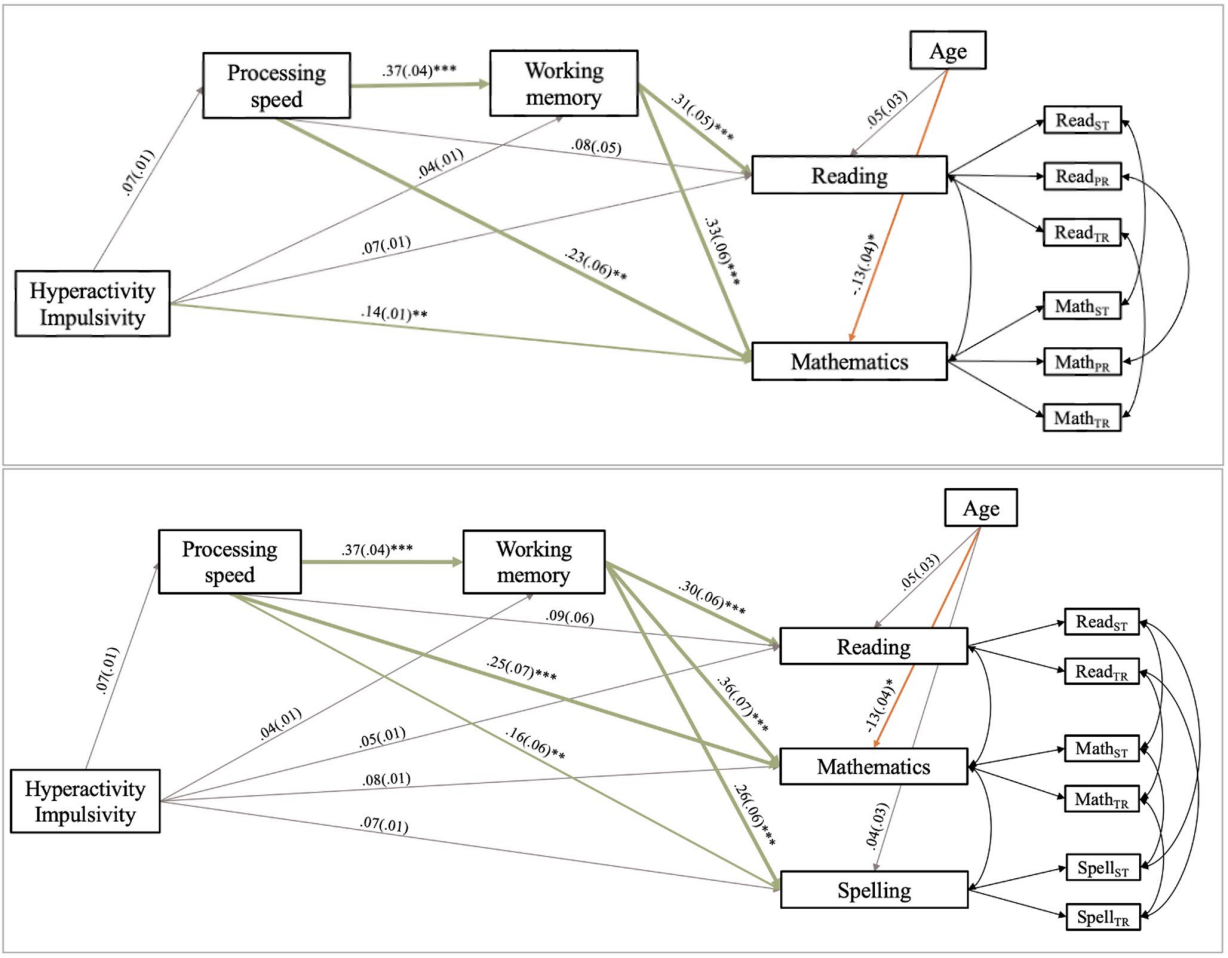
Visual representation of serial mediation model 1 (top) and serial mediation model 2 (bottom) for hyperactivity/impulsivity symptom severity as predictor. *Note. *ST = standardized test, TR = teacher rating, PR = parent rating

Findings of both measurement models indicated good model fit, with good convergent validity for the different academic subjects. The discriminant validity between reading and spelling, however, was poor as a high correlation between both factors was found. To account for the poor discriminant validity, as well as controlling for measurement method effects, two serial mediation models were fitted: serial mediation model 1 with measurement model 1 fully implemented and serial mediation model 2 with measurement model 2 fully implemented. Thus, multiple academic subjects were included simultaneously in both serial mediation models (see Figs. 3 and 4).

As the models were run separately for symptoms of inattention and hyperactivity/impulsivity, four final serial mediation models were fitted. All four models showed good fit to the data, as shown by the model fit indices (see Table 6). The standardized beta coefficients can be found in Figs. 3 and 4, and the indirect effects of the different serial mediation models can be found in Table 6. Results for the models fitted for verbal WM and visual-spatial WM separately are reported when relevant and different from the main analyses. Full results are reported in Supplement B.

**Table 6:** Fit indices, standardized beta coefficients for direct effects and unstandardized indirect effects with 95% bootstrap confidence intervals for the serial mediation models as shown in Fig. 3

	Inattention		Hyperactivity/Impulsivity	
	Model 1	Model 2	Model 1	Model 2
Model fit				
χ² (df)	34.54 (23)	28.18 (17)	46.412(23)	34.83 (17)
CFI	0.99	0.99	0.98	0.99
RMSEA	0.03	0.04	0.05	0.05
SMSR	0.02	0.03	0.03	0.03
*Single indirect pathways* [95% bootstrap confidence intervals]				
PS – Reading	-.001 [-.004, .001]	-.001 [-.004, .0004]	.001 [-.0003, .003]	.001 [-.0004, .004]
PS – Mathematics	-.004 [-.010, -.0001]*	-.005 [-.011, -.0001]*	.003 [-.001, .008]	.003 [-.001, .008]
PS - Spelling		-.003 [-.006, -.00001]*		.002 [-.001, .005]
WM – Reading	-.001 [-.006, .004]	-.001 [-.006, .004]	.002 [-.002, .006]	.002 [-.002, .006]
WM – Mathematics	-.001 [-.007, .005]	-.001 [-.008, .005]	.002 [-.002, .007]	.003 [-.003, .008]
WM - Spelling		-.001 [-.005, .004]		.002 [-.002, .005]
PS – WM	-.006 [-.012, -.0001]*	-.006 [-.012, -.0002]*	.004 [-.001, .010]	.004 [-.001, .010]
*Serial indirect pathways *[95% bootstrap confidence intervals]				
PS – WM – Reading	-.002 [-.004, -.00003]*	-.002 [-.004, -.00005]*	.001 [-.0004, .003]	.001 [-.0004, .003]
PS – WM - Mathematics	-.002 [-.005, -.00004]*	-.003 [-.006, -.0001]*	.002 [-.0005, .004]	.002 [-.0004, .005]
PS – WM – Spelling		-.002 [-.004, -.00004]*		.001 [-.0003, .003]

*p <.05, **p <.01, ***p <.001

For the CI or estimators of the indirect pathways, more decimals are displayed to indicate whether the estimate is different from zero or the CI interval contains the value zero

#### Reading

##### Inattention Symptom Severity

The direct effect between inattention symptom severity and reading performance was significant in both serial mediation models. The single indirect effect with PS as mediator between inattention symptom severity and reading performance was non-significant for both serial mediation models. The single indirect effect with WM as mediator between inattention symptom severity and reading performance was also not significant for both serial mediation models. Thus, neither PS, nor WM was a (partial) mediator in the relationship between inattention symptom severity and reading performance (see Table 6).

The direct effect between inattention symptom severity and WM, as well as the indirect effect between both through PS, is the same for both serial mediation model 1 and model 2. The direct effect between inattention symptom severity and WM was not significant, but the indirect effect through PS was significant. This indicates that the relationship between inattention symptom severity and WM performance is fully mediated through PS (see Table 6).

Moreover, the serial mediation effect through both PS and WM was significant for both models. Given that the direct pathway between inattention symptom severity and reading performance was significant for both serial mediation model 1 and model 2, these results indicate this relation is partially mediated through both PS and WM (see Table 6). The single and serial mediation effects were not significant for the models with verbal WM and visual-spatial WM as separate indicators of WM performance (see Table S15 and S16).

##### Hyperactivity/Impulsivity Symptom Severity

The direct effect between hyperactivity/impulsivity symptom severity and reading performance was not significant in both serial mediation models. None of the single indirect pathways or the serial indirect pathway was significant in these models, indicating that this relationship between hyperactivity/impulsivity symptom severity is not mediated through PS and/or WM for both models (see Table 6).

##### Age

Across both serial mediation models, and for both inattention and hyperactivity/impulsivity symptom severity, age was not a significant predictor of reading performance.

#### Mathematics

##### Inattention Symptom Severity

The direct effect between inattention symptom severity and mathematics performance was significant in serial mediation model 1, but not significant in serial mediation model 2. The single indirect effect of PS as mediator between inattention symptom severity and mathematics performance was significant for both serial mediation models. Thus, PS was a partial mediator in the relationship between inattention symptom severity and mathematics performance in mediation model 1, and a full mediator in mediation model 2. The single indirect effect with WM as mediator in the relation between inattention symptom severity and mathematics performance was not significant in both serial mediation models. Thus, WM was not a (partial) mediator in the relationship between inattention symptom severity and mathematics performance. As mentioned above, PS fully mediated the relationship between inattention symptom severity and WM performance (Table 6).

The serial mediation effects through PS and WM were also significant for mathematics performance across both models. The direct pathway from inattention symptom severity to mathematics performance was significant for serial mediation model 1, but not for serial mediation model 2, and thus PS and WM respectively partially and fully mediated the relation between both (Table 6). None of the single or serial indirect effects were significant in the models with verbal WM and visual-spatial WM included separately (see Table S15 and S16).

##### Hyperactivity/Impulsivity Symptom Severity

The direct effect between hyperactivity/impulsivity symptom severity and mathematics performance was significant in serial mediation model 1, but not in serial mediation model 2. None of the single indirect pathways, nor the serial indirect pathway were significant when hyperactivity/impulsivity symptom severity scores were inserted as predictor variable. This indicates hyperactivity/impulsivity symptom severity significantly predicts mathematics performance, but this relation is not mediated through PS or WM (Table 6).

##### Age

In all four serial mediation models, the effect of age was significant. Older children showed poorer mathematics performance compared to younger children.

#### Spelling

As spelling was not included in serial mediation model 1, all reported results for spelling are retrieved from serial mediation model 2.

##### Inattention Symptom Severity

The direct effect between inattention symptom severity and spelling performance was significant. The single indirect effect of PS as mediator in the relation between inattention symptom severity and spelling performance was significant. The single indirect effect of WM as mediator in the relation between inattention symptom severity and spelling performance was not significant. Thus, PS, but not WM was a partial mediator in the relationship between inattention symptom severity and spelling performance. As mentioned earlier, the effect between inattention symptom severity and WM performance was fully mediated through PS (Table 6).

The serial mediation effect through PS and WM was also significant in the relation between inattention symptom severity and spelling performance. Thus, given the direct effect between inattention symptom severity and spelling performance was significant, these results indicate this relation is partially mediated by PS and WM (Table 6). None of the single or serial indirect effects were significant in the models with verbal WM and visual-spatial WM included separately (see Table S15 and S16).

##### Hyperactivity/Impulsivity Symptom Severity

The direct effect between hyperactivity/impulsivity symptom severity and spelling performance was not significant. None of the single indirect pathways, nor the serial indirect pathway was significant in the relation between hyperactivity/impulsivity symptom severity and spelling performance. Thus, hyperactivity/impulsivity symptom severity scores are not a significant predictor of spelling performance, and this relation is not mediated through PS and/or WM.

##### Age

Neither for the model with inattention symptom severity, nor for hyperactivity/impulsivity symptom severity, age was a significant predictor of spelling performance.

#### Sex

Equality constraints were imposed on direct and indirect pathways to investigate whether they could be equalized across male and female participants, and thus whether effects could be interpreted the same way for both sex groups. Results show that (in)direct effects could be equalized across both groups, and thus effects were sufficiently similar for male and female participants (Table 7).

**Table 7:** Chi-square values and chi-square difference test for the unrestricted and restricted model investigating model invariance across male and female participants

	Chi-square	Df	Chi-square difference	Df difference	*p*
*Serial mediation model 1 – Inattention symptoms*					
Unrestricted	123.25	64			
Restricted	135.10	75	11.84	11	0.376
*Serial mediation model 1 – Hyperactivity/impulsivity symptoms*					
Unrestricted	82.15	50			
Restricted	99.40	65	17.25	15	0.304
*Serial mediation model 2 – Inattention symptoms*					
Unrestricted	130.56	64			
Restricted	142.30	75	11.74	11	0.384
*Serial mediation model 2 – Hyperactivity/impulsivity symptoms*					
Unrestricted	94.32	50			
Restricted	110.97	65	16.65	15	0.340

## Discussion

The current study is the first to investigate the role of both PS and WM in the relation between ADHD symptom severity and academic achievement, measured for multiple academic subjects, across multiple measurement methods, in children with ADHD. Our results showed that standardized tests, parent ratings and teacher ratings for the different academic subjects showed good convergent validity, also when controlling for measurement method effects. Our most important finding is the importance of PS in academic achievement for children with inattention symptoms; the relation between inattention symptom severity, and reading, mathematics, and spelling performance was statistically mediated by PS and WM sequentially. For mathematics and spelling performance, but not for reading, the single mediating effect of PS was also statistically significant. PS also statistically mediated the relation between inattention symptom severity and WM performance. Hyperactive/impulsive symptom severity scores were positively related to mathematics performance, but none of the (serial) mediation effects were significant for this symptom domain of ADHD. Lastly, age negatively predicted mathematics performance, but did not predict reading or spelling performance.

The relation between inattention symptom severity and reading, mathematics or spelling performance was statistically mediated serially through PS and WM. This is in line with recent studies, indicating that deficits in more basic cognitive skills, such as PS, may underly poorer WM, contributing to academic impairments (Braaten et al., 2020; Lee, 2024; Rose et al., 2011). Importantly, although results mostly overlap between the two mediation models used for reading and mathematics, the effect was only partially mediated for most models, but full mediation emerged for mathematics performance when parent ratings of academic achievement were not included. This indicates that parent ratings of academic achievement add certain variability to the measurement of mathematics performance that cannot be explained by the included cognitive functions. Parents for example, may consider homework behavior in their rating of academic achievement, behavior that is often shown to be disrupted in children with ADHD (Power et al., 2006). These behavioral difficulties may not be explained by processing speed and working memory. In addition, classical classroom structures often cause different types of behavior compared to the home context, leading to different assessment of academic performance by the teachers. For example, more distraction may be present in a classroom context, and less individual feedback or support is available. Future studies should investigate the role of other variables in this relation, to elaborate further on this association.

The results of our study are also in line with previous studies, indicating that a more general cognitive deficit in PS may underly poorer WM performance in children with ADHD (Karalunas & Huang-Pollock, 2013; Weigard & Huang-Pollock, 2017). It also provides support for the TBRS model, hypothesizing slower PS causes an increased cognitive load, causing WM decay (Portrat et al., 2009). However, on contrast to earlier studies (Karalunas & Huang-Pollock, 2013), our results show the relation between inattention symptom severity and WM is statistically fully mediated by PS. A potential explanation for these differences in results lay in the operationalization of the variables as used. Whereas previous studies used ADHD status (i.e., a binary variable) as predictor in the model, we implemented ADHD symptom severity scores, potentially resulting in more variance to be explained (Karalunas & Huang-Pollock, 2013). Moreover, a different operationalization of PS was used across both studies, as our results are based on the PS index score from the WISC-IV or WISC-V (Wechsler, 2003, 2014). In these subtests, children need to complete as many items correctly as possible in the provided time. The previous study on the other hand, retrieved PS from a Stop Signal Reaction time task, based on the response time of the child for each trial (Karalunas & Huang-Pollock, 2013). Research shows different measures of PS tap into different aspects of the cognitive function (Frazier et al., 2004).

The single mediation effect, with PS as mediator, was statistically significant for the relation between inattention symptoms and both mathematics and spelling performance, but not for reading performance. This may suggest that different academic subjects have different cognitive predictors. Research for example shows that different cognitive functions underly reading and spelling (Vaessen & Blomert, 2013). This highlights the importance of investigating specific contributing factors to underachievement in different academic subjects in children with ADHD in future studies.

The multitrait-multimethod models as applied for the current study, permits testing of convergent validity, considering the effects of the different measurement methods. Results of our study showed good convergent validity emerged for the different academic subjects while adjusting for method effects. This indicates that standardized tests, parent ratings, and teacher ratings significantly relate to each other within each academic subject, considering the method effects of each measurement instrument. Our results support the validity of combining data from multiple methods and sources to provide a more complete perspective of the concept investigated (i.e., *triangulation*; Moon, 2019), in this case for children’s academic achievement.

However, discriminant validity between reading and spelling performance was poor, as a high correlation between both factors emerged. Nonetheless, comparing the three-factor model (i.e., reading, mathematics and spelling) to a two-factor model, where reading and spelling scores were collapsed into one factor, showed that model fit was significantly better for the three-factor model. Moreover, modification indices of the two-factor model indicate the two measures for reading, as well as the two measures for spelling, share unique variance that could not be explained by a more general factor, supporting the distinction between reading and spelling in our mediation models. Additionally, research shows reading and spelling have different developmental paths and their cognitive contributors diverge (Vaessen & Blomert, 2013), providing support for the decision to distinguish between both factors.

The significant statistical mediation effects found across the models, were not significant when verbal WM and visual-spatial WM were inserted separately. A potential explanation for this lack of significance, is the reduced variability in WM performance scores when considering only one subtest instead of the composite score of two subtests. As shown in Table 1, the standard deviations for each of the two WM subtests is significantly lower compared to the standard deviation of the combined index score. As a result, statistical variability is lower for these subtests, thereby reducing the amount of variance available to explain statistically. Additionally, verbal WM or visual-spatial WM separately may not have enough predictive value regarding academic performance. Another explanation for the lack of significant results when considering the two different WM subtests, is the reduced sample size in these analyses (*n* = 369), compared to the full sample (*n* = 504). In SEM analyses, sample size affects the precision of all statistical estimates, potentially influencing the results. However, common rules of thumb indicate necessary sample sizes of *n* = 300 or even *n* = 200 for SEM, suggesting the power of the reduced sample size in our study is still sufficient to find effect (Kyriazos, 2018a). Future studies should further investigate the contribution of both aspects of WM in relation to academic achievement.

The statistical serial mediation of PS and WM in the relation between inattention symptom severity and academic achievement, may have important implications for both theoretical accounts, as well as interventions for academic problems in children with ADHD. WM deficits are often assumed to be contributing to ADHD symptomatology, thereby causing functional impairments. Our results suggest the importance of considering PS as well, beyond focusing on WM, when considering academic difficulties in this population. Future studies would benefit from including measures of PS, when investigating cognitive predictors of academic performance in ADHD. Additionally, interventions for academic underperformance in ADHD often target WM deficits (Daley & Birchwood, 2010; Martinussen & Major, 2011). This study highlights the importance of considering PS as well, potentially complementary to targeting WM. Children with ADHD can for example be provided with additional time to process assignments and tests or delivered important information or course content in advance to allow them more time to prepare and process certain materials.

The mostly non-significant effects of hyperactivity/impulsivity symptom severity as a predictor is consistent with previous research, indicating it is primarily symptoms of inattention that play a significant role in academic achievement (Garner et al., 2013). Surprisingly however, hyperactivity/impulsivity symptom severity significantly predicted mathematics performance, with higher symptom severity scores related to better performance. The state regulation deficit (SRD) theory may offer a potential explanation for these results. According to this theory, children with ADHD are generally more often under-aroused, which causes them to underperform on cognitive tasks. The hyperactive behavior of these children is assumed to be a compensatory mechanism to optimize their arousal levels, thereby improving their task performance (Kofler et al., 2016). Potentially, children in the current sample who showed increased parent-reported hyperactive behavior, can regulate their energetic state through movement, therefore optimizing their academic performance. However, this should be further investigated experimentally by examining the effects of movement during academic tasks. A possible alternative explanation is that children displaying elevated symptoms of hyperactivity/impulsivity, are potentially more likely to receive pharmacotherapy for their ADHD symptoms, compared to those showing primarily inattentive symptoms. Although all children taking stimulants were medication free during research participation, being medicated likely increases their effective engagement in the classroom, improving their overall academic performance.

Lastly, age was a significant predictor of mathematics performance in the current study, with older children showing lower academic performance scores. The standardized test scores were based on grade specific norms, and parent and teacher ratings were performed in comparison with peers of the same age. This suggests older children with inattention symptoms show increased academic underperformance, relative to their peers, especially with regards to mathematics. These results are in line with previous studies showing a similar effect (Murray et al., 2017). Contrary to previous studies, however, where a significant subgroup of the total sample showed a decline across all three academic subjects (Murray et al., 2017), this effect was not significant for reading and spelling performance. Children in the current study were younger, and the previous study was longitudinal, allowing within-subject comparisons over time, whereas we performed cross-sectional between-subject comparisons.

The current findings may also have implications for research in other clinical populations, or other co-occurring conditions in ADHD. For example, the role of both processing speed (Stenneken et al., 2011) and working memory (Gray et al., 2019; Szucs et al., 2013) in learning disorders, such as dyslexia, dyscalculia, and developmental language disorders, has been well established. However, to our knowledge, there is a lack of studies investigating potential mediation effects in relation to these conditions, and future studies would benefit integrating both cognitive functions as it may give indications to optimize interventions.

The current study is not without limitations. First, given the cross-sectional and observational study design, no causal inferences can be made about the current results, and one could argue against the assumed directionality of effects. It could be argued that reduced WM performance reduces speed of information processing, thereby impacting academic functioning. However, when we tested such a model, i.e., where WM predicts PS in the serial mediation, none of the mediation effects as reported earlier remained significant (see Supplement D). In addition, a previous study has found the mediation of PS and WM to be significant when longitudinally investigated, but in premature children and not children with ADHD (Cassidy et al., 2016). An experimental study in children with ADHD also found PS to be a significant mediator in the relation with WM, but the relation with academic achievement was not investigated (Weigard & Huang-Pollock, 2017). Future studies should investigate the serial mediation of PS and WM in the relation between ADHD symptoms and academic achievement in a longitudinal or experimental study. Second, the estimation method used for the current study was Maximum Likelihood (ML), which is shown to be reliable, but potentially leading to biased parameter estimates when ordinal variables with 4 response categories are included in the model (Li, 2016). As parent ratings of academic performance were measured on a 4-point rating scale, the parameter estimates of our models including parent ratings should be interpreted with caution. However, our results appear to be robust, as the serial mediation effects were also statistically significant for the second mediation model, that didn’t include these parent ratings. Next, some of the subtests to measure academic performance (Word reading, Math computation and Spelling from the WRAT-4) include time limits, providing a potential explanation for the relationship between PS and academic performance. However, the subtests from the other standardized tests were not timed (WIAT-3 and WIAT-4) and measurement invariance was proven across the different test versions used. In addition, academic performance was also measured via parent and teacher ratings, indicating the link is not primarily explained by the timing limitation of these few subtests. Lastly, the investigated sample primarily comprised of US citizens temporarily residing in Japan and most of the participants where either Caucasian or had a mixed racial background (see Table 1). This may limit the generalizability of the results and future research should replicate current findings across different cultural groups.

## Conclusion

In the current study, the serial mediation role of PS and WM was investigated in the relation between ADHD symptom severity and reading, mathematics and spelling performance. The findings add to the current available evidence, showing that PS plays an important role in academic underachievement in children with ADHD, potentially underlying WM deficits as demonstrated in these children (Sowerby et al., 2011). These results have important implications for theoretical accounts on executive functioning in ADHD, which often include WM deficits (Martinussen et al., 2005; Sonuga-Barke, 2003), and highlight the importance for future research to investigate the relation between both. Moreover, it suggests academic interventions for children with ADHD may include strategies to accommodate for a slower PS, as they currently primarily target WM deficits (Daley & Birchwood, 2010; Martinussen & Major, 2011).

## Electronic Supplementary Material

Below is the link to the electronic supplementary material.

Supplementary Material 1

Supplementary Material 2

Supplementary Material 3

Supplementary Material 4

## Data Availability

The first and last authors have access to all supporting data. As the data is not anonymously archived it is not currently openly available. Research requests for data access should be directed to the last author.

## References

[CR1] Alloway, T. P., & Alloway, R. G. (2010). Investigating the predictive roles of working memory and IQ in academic attainment. *Journal of Experimental Child Psychology*, *106*(1), 20–29. 10.1016/j.jecp.2009.11.003 20018296 10.1016/j.jecp.2009.11.003

[CR2] American Psychiatric Association (2022). *Diagnostic and statistical manual of mental disorders* (DSM-5-TR). American Psychiatric Association Publishing. 10.1176/appi.books.9780890425787

[CR3] Arnold, L. E., Hodgkins, P., Kahle, J., Madhoo, M., & Kewley, G. (2020). Long-term outcomes of ADHD: Academic achievement and performance. *Journal of Attention Disorders*, *24*(1), 73–85. 10.1177/1087054714566076 25583985 10.1177/1087054714566076

[CR4] Barbaresi, W. J., Katusic, S. K., Colligan, R. C., Weaver, A. L., & Jacobsen, S. J. (2007). Long-term school outcomes for children with attention-deficit/hyperactivity disorder: A population-based perspective. *Journal of Developmental & Behavioral Pediatrics*, *28*(4), 265–273. 10.1097/DBP.0b013e31811ff87d 17700078 10.1097/DBP.0b013e31811ff87d

[CR5] Braaten, E. B., Ward, A. K., Forchelli, G., Vuijk, P. J., Cook, N. E., McGuinness, P., Lee, B. A., Samkavitz, A., Lind, H., O’Keefe, S. M., & Doyle, A. E. (2020). Characteristics of child psychiatric outpatients with slow processing speed and potential mechanisms of academic impact. *European Child & Adolescent Psychiatry*, *29*(10), 1453–1464. 10.1007/s00787-019-01455-w 31980930 10.1007/s00787-019-01455-w PMC8168921

[CR6] Brown, T. A. (2006). *Confirmatory factor analysis for applied research*. Guilford Press.

[CR7] Caemmerer, J. M., Maddocks, D. L. S., Keith, T. Z., & Reynolds, M. R. (2018). Effects of cognitive abilities on child and youth academic achievement: Evidence from the WISC-V and WIAT-III. *Intelligence*, *68*, 6–20. 10.1016/j.intell.2018.02.005

[CR8] Cassidy, A. R., White, M. T., DeMaso, D. R., Newburger, J. W., & Bellinger, D. C. (2016). Processing speed, executive function, and academic achievement in children with dextro-transposition of the great arteries: Testing a longitudinal developmental cascade model. *Neuropsychology*, *30*(7), 874–885. 10.1037/neu0000289 27077787 10.1037/neu0000289 PMC5042819

[CR9] Conners, C. K. (2008). *Conners comprehensive behavior rating scales: Manual*. Multi-Health systems.

[CR10] Cook, N. E., Braaten, E. B., & Surman, C. B. H. (2018). Clinical and functional correlates of processing speed in pediatric attention-deficit/hyperactivity disorder: A systematic review and meta-analysis. *Child Neuropsychology*, *24*(5), 598–616. 10.1080/09297049.2017.1307952 28345402 10.1080/09297049.2017.1307952

[CR11] R Core Team (2024). *A language and environment for statistical computing.* (Version 4.3.3) [Computer software]. R Foundation for Statistical Computing. https://www.R-project.org/

[CR12] Daley, D., & Birchwood, J. (2010). ADHD and academic performance: Why does ADHD impact on academic performance and what can be done to support ADHD children in the classroom? *Child: Care Health and Development*, *36*(4), 455–464. 10.1111/j.1365-2214.2009.01046.x 20074251 10.1111/j.1365-2214.2009.01046.x

[CR13] Dodonova, Y. A., & Dodonov, Y. S. (2012). Processing speed and intelligence as predictors of school achievement: Mediation or unique contribution? *Intelligence*, *40*(2), 163–171. 10.1016/j.intell.2012.01.003

[CR14] Faraone, S. V., Banaschewski, T., Coghill, D., Zheng, Y., Biederman, J., Bellgrove, M. A., Newcorn, J. H., Gignac, M., Al Saud, N. M., Manor, I., Rohde, L. A., Yang, L., Cortese, S., Almagor, D., Stein, M. A., Albatti, T. H., Aljoudi, H. F., Alqahtani, M. M. J., Asherson, P., & Wang, Y. (2021). The world federation of ADHD international consensus statement: 208 evidence-based conclusions about the disorder. *Neuroscience & Biobehavioral Reviews*, *128*, 789–818. 10.1016/j.neubiorev.2021.01.022 33549739 10.1016/j.neubiorev.2021.01.022 PMC8328933

[CR15] Fleming, M., Fitton, C. A., Steiner, M. F. C., McLay, J. S., Clark, D., King, A., Mackay, D. F., & Pell, J. P. (2017). Educational and health outcomes of children treated for attention-deficit/hyperactivity disorder. *JAMA Pediatrics*, *171*(7), e170691. 10.1001/jamapediatrics.2017.0691 28459927 10.1001/jamapediatrics.2017.0691 PMC6583483

[CR16] Frazier, T. W., Demaree, H. A., & Youngstrom, E. A. (2004). Meta-analysis of intellectual and neuropsychological test performance in attention-deficit/hyperactivity disorder. *Neuropsychology*, *18*(3), 543–555. 10.1037/0894-4105.18.3.543 15291732 10.1037/0894-4105.18.3.543

[CR17] Frazier, T. W., Youngstrom, E. A., Glutting, J. J., & Watkins, M. W. (2007). ADHD and achievement: Meta-analysis of the child, adolescent, and adult literatures and a concomitant study with college students. *Journal of Learning Disabilities*, *40*(1), 49–65. 10.1177/00222194070400010401 17274547 10.1177/00222194070400010401

[CR18] Garner, A. A., O’Connor, B. C., Narad, M. E., Tamm, L., Simon, J., & Epstein, J. N. (2013). The relationship between ADHD symptom dimensions, clinical correlates, and functional impairments. *Journal of Developmental & Behavioral Pediatrics*, *34*(7), 469–477. 10.1097/DBP.0b013e3182a39890 24042078 10.1097/DBP.0b013e3182a39890 PMC4014057

[CR19] Geary, D. C. (2011). Cognitive predictors of achievement growth in mathematics: A 5-year longitudinal study. *Developmental Psychology*, *47*(6), 1539–1552. 10.1037/a0025510 21942667 10.1037/a0025510 PMC3210883

[CR20] Gnanavel, S., Sharma, P., Kaushal, P., & Hussain, S. (2019). Attention deficit hyperactivity disorder and comorbidity: A review of literature. *World Journal of Clinical Cases*, *7*(17), 2420–2426. 10.12998/wjcc.v7.i17.2420 31559278 10.12998/wjcc.v7.i17.2420 PMC6745333

[CR21] Goth-Owens, T. L., Martinez-Torteya, C., Martel, M. M., & Nigg, J. T. (2010). Processing speed weakness in children and adolescents with non-hyperactive but inattentive ADHD (ADD). *Child Neuropsychology*, *16*(6), 577–591. 10.1080/09297049.2010.485126 20560083 10.1080/09297049.2010.485126 PMC2943531

[CR22] Gray, S., Fox, A. B., Green†, S., Alt, M., Hogan, T. P., Petscher, Y., & Cowan, N. (2019). Working memory profiles of children with dyslexia, developmental language disorder, or both. *Journal of Speech Language and Hearing Research*, *62*(6), 1839–1858. 10.1044/2019_JSLHR-L-18-0148 10.1044/2019_JSLHR-L-18-0148 PMC6808376 31112436

[CR23] Greenhill, L. L., Pliszka, S., & Dulcan, M. K. (2002). Practice parameter for the use of stimulant medications in the treatment of children, adolescents, and adults. *Journal of the American Academy of Child & Adolescent Psychiatry*, *41*(2), 26S–49. 10.1097/00004583-200202001-00003. S. 11833633 10.1097/00004583-200202001-00003

[CR24] Karalunas, S. L., & Huang-Pollock, C. L. (2013). Integrating impairments in reaction time and executive function using a diffusion model framework. *Journal of Abnormal Child Psychology*, *41*(5), 837–850. 10.1007/s10802-013-9715-2 23334775 10.1007/s10802-013-9715-2 PMC3679296

[CR25] Kaufman, J., Birmaher, B., Brent, D., Rao, U., Flynn, C., Moreci, P., Williamson, D., Ryan, N., Version, L., & K-SADS-PL. (1997). (): Initial reliability and validity data. *Journal of the American Academy of Child & Adolescent Psychiatry*, 36(7), 980–988. 10.1097/00004583-199707000-00021. 9204677 10.1097/00004583-199707000-00021

[CR26] Kaufman, J., Birmaher, B., Axelson, D., Pereplitchikova, F., Brent, D., & Ryan, N. (2016). *The KSADS-PL DSM-5*. Kennedy Krieger Institute.

[CR27] Kent, K. M., Pelham, W. E., Molina, B. S. G., Sibley, M. H., Waschbusch, D. A., Yu, J., Gnagy, E. M., Biswas, A., Babinski, D. E., & Karch, K. M. (2011). The academic experience of male high school students with ADHD. *Journal of Abnormal Child Psychology*, *39*(3), 451–462. 10.1007/s10802-010-9472-4 21103923 10.1007/s10802-010-9472-4 PMC3068222

[CR28] Kibby, M. Y., Vadnais, S. A., & Jagger-Rickels, A. C. (2019). Which components of processing speed are affected in ADHD subtypes? *Child Neuropsychology*, *25*(7), 964–979. 10.1080/09297049.2018.1556625 30558479 10.1080/09297049.2018.1556625 PMC6581645

[CR29] Kofler, M. J., Raiker, J. S., Sarver, D. E., Wells, E. L., & Soto, E. F. (2016). Is hyperactivity ubiquitous in ADHD or dependent on environmental demands? Evidence from meta-analysis. *Clinical Psychology Review*, *46*, 12–24. 10.1016/j.cpr.2016.04.004 27131918 10.1016/j.cpr.2016.04.004 PMC4902796

[CR30] Kyriazos, T. A. (2018a). Applied psychometrics: sample size and sample power considerations in factor analysis (EFA, CFA) and SEM in general. *Psychology*, *09*(08), 2207–2230. 10.4236/psych.2018.98126

[CR31] Kyriazos, T. A. (2018b). Applied psychometrics: The application of CFA to Multitrait-Multimethod matrices (CFA-MTMM). *Psychology*, *09*(12), 2625–2648. 10.4236/psych.2018.912150

[CR32] Lee, C. S. C. (2024). Processing speed deficit and its relationship with math fluency in children with attention-deficit/hyperactivity disorder. *Journal of Attention Disorders*, *28*(2), 211–224. 10.1177/10870547231211022 37981794 10.1177/10870547231211022

[CR33] Li, C. H. (2016). Confirmatory factor analysis with ordinal data: Comparing robust maximum likelihood and diagonally weighted least squares. *Behavior Research Methods*, *48*(3), 936–949. 10.3758/s13428-015-0619-7 26174714 10.3758/s13428-015-0619-7

[CR34] Martinussen, R., & Major, A. (2011). Working memory weaknesses in students with ADHD: Implications for instruction. *Theory into Practice*, *50*(1), 68–75. 10.1080/00405841.2011.534943

[CR35] Martinussen, R., Hayden, J., Hogg-Johnson, S., & Tannock, R. (2005). A meta-analysis of working memory impairments in children with attention-deficit/hyperactivity disorder. *Journal of the American Academy of Child & Adolescent Psychiatry*, *44*(4), 377–384. 10.1097/01.chi.0000153228.72591.73 15782085 10.1097/01.chi.0000153228.72591.73

[CR36] Moon, M. D. (2019). Triangulation: A method to increase validity, reliability, and legitimation in clinical research. *Journal of Emergency Nursing*, *45*(1), 103–105. 10.1016/j.jen.2018.11.004 30616761 10.1016/j.jen.2018.11.004

[CR37] Murray, A. L., Robinson, T., & Tripp, G. (2017). Neurocognitive and symptom trajectories of ADHD from childhood to early adolescence. *Journal of Developmental & Behavioral Pediatrics*, *38*(7), 465–475. 10.1097/DBP.0000000000000476 28723827 10.1097/DBP.0000000000000476

[CR38] Portrat, S., Camos, V., & Barrouillet, P. (2009). Working memory in children: A time-constrained functioning similar to adults. *Journal of Experimental Child Psychology*, *102*(3), 368–374. 10.1016/j.jecp.2008.05.005 18632113 10.1016/j.jecp.2008.05.005

[CR39] Power, T. J., Werba, B. E., Watkins, M. W., Angelucci, J. G., & Eiraldi, R. B. (2006). Patterns of parent-reported homework problems among ADHD-referred and non-referred children. *School Psychology Quarterly*, *21*(1), 13–33. 10.1521/scpq.2006.21.1.13

[CR40] Preacher, K. J., & Hayes, A. F. (2008). Asymptotic and resampling strategies for assessing and comparing indirect effects in multiple mediator models. *Behavior Research Methods*, *40*(3), 879–891. 10.3758/BRM.40.3.879 18697684 10.3758/brm.40.3.879

[CR41] Rose, S. A., Feldman, J. F., & Jankowski, J. J. (2011). Modeling a cascade of effects: The role of speed and executive functioning in preterm/full-term differences in academic achievement: Executive function in preterm adolescents. *Developmental Science*, *14*(5), 1161–1175. 10.1111/j.1467-7687.2011.01068.x 21884331 10.1111/j.1467-7687.2011.01068.x

[CR42] Simone, A. N., Marks, D. J., Bédard, A. C., & Halperin, J. M. (2018). Low working memory rather than ADHD symptoms predicts poor academic achievement in school-aged children. *Journal of Abnormal Child Psychology*, *46*(2), 277–290. 10.1007/s10802-017-0288-3 28357519 10.1007/s10802-017-0288-3 PMC5620112

[CR43] Sjöwall, D., & Thorell, L. B. (2014). Functional impairments in attention deficit hyperactivity disorder: The mediating role of neuropsychological functioning. *Developmental Neuropsychology*, *39*(3), 187–204. 10.1080/87565641.2014.886691 24742310 10.1080/87565641.2014.886691 PMC4017272

[CR44] Sonuga-Barke, E. J. S. (2003). The dual pathway model of AD/HD: An elaboration of neuro-developmental characteristics. *Neuroscience & Biobehavioral Reviews*, *27*(7), 593–604. 10.1016/j.neubiorev.2003.08.005 14624804 10.1016/j.neubiorev.2003.08.005

[CR45] Sowerby, P., Seal, S., & Tripp, G. (2011). Working memory deficits in ADHD: The contribution of age, learning/language difficulties, and task parameters. *Journal of Attention Disorders*, *15*(6), 461–472. 10.1177/1087054710370674 20574057 10.1177/1087054710370674

[CR46] Stenneken, P., Egetemeir, J., Schulte-Körne, G., Müller, H. J., Schneider, W. X., & Finke, K. (2011). Slow perceptual processing at the core of developmental dyslexia: A parameter-based assessment of visual attention. *Neuropsychologia*, *49*(12), 3454–3465. 10.1016/j.neuropsychologia.2011.08.021 21903119 10.1016/j.neuropsychologia.2011.08.021

[CR47] Swanson, J. M., Schuck, S., Porter, M. M., Carlson, C., Hartman, C. A., Sergeant, J. A., Clevenger, W., Wasdell, M., McCleary, R., Lakes, K., & Wigal, T. (2012). Categorical and dimensional definitions and evaluations of symptoms of ADHD: History of the SNAP and the SWAN rating scales. *The International Journal of Educational and Psychological Assessment*, *10*(1), 51–70. 26504617 PMC4618695

[CR48] Szucs, D., Devine, A., Soltesz, F., Nobes, A., & Gabriel, F. (2013). Developmental dyscalculia is related to visuo-spatial memory and Inhibition impairment. *Cortex; A Journal Devoted To the Study of the Nervous System and Behavior*, *49*(10), 2674–2688. 10.1016/j.cortex.2013.06.007 23890692 10.1016/j.cortex.2013.06.007 PMC3878850

[CR49] Vaessen, A., & Blomert, L. (2013). The cognitive linkage and divergence of spelling and reading development. *Scientific Studies of Reading*, *17*(2), 89–107. 10.1080/10888438.2011.614665

[CR50] Wechsler, D. (2003). *Wechsler intelligence scale for children, fourth edition (WISC-IV)*. The Psychological Corporation.

[CR51] Wechsler, D. (2009). *Wechsler individual achievement test* (3rd ed.).). Psychological Corporation.

[CR52] Wechsler, D. (2014). *Wechsler intelligence scale for children—Fifth edition (WISC-V)*. Pearson.

[CR53] Wechsler. (2020). *Wechsler individual achievement test (* (4th ed.).). Psychological Corporation.

[CR54] Weigard, A., & Huang-Pollock, C. (2017). The role of speed in ADHD-related working memory deficits: A time-based resource-sharing and diffusion model account. *Clinical Psychological Science*, *5*(2), 195–211. 10.1177/2167702616668320 28533945 10.1177/2167702616668320 PMC5437983

[CR55] Wilkinson, G. S., & Robertson, G. J. (2006). *Wide range achievement test, fourth edition (WRAT-4)*. Psychological Assessment Resources, Inc.

