# Supplementary material for: Academic Achievement in Children with ADHD: the Role of Processing Speed and Working Memory
Source: Res Child Adolesc Psychopathol. 2025 Jul 15;53(10):1469–84. doi: 10.1007/s10802-025-01346-6 (PMC12521309; doi:10.1007/s10802-025-01346-6)
Supplement: Supplementary file 1 — Supplementary Material 1 [file 10802_2025_1346_MOESM1_ESM.docx]

**Title:** Academic achievement in children with ADHD: The role of processing speed and working memory

**Journal name:** Research on Child and Adolescent Psychopathology

**Author names:** An-Katrien Hulsbosch, Saskia Van der Oord* & Gail Tripp* (*joint last authors)

**Corresponding author:** An-Katrien Hulsbosch

**Supplement A. Measurement invariance tests for the different administered versions of standardized tests and questionnaires used in the different fitted models.**

Multiple variables as included in the models fitted for the current study, were measured through different versions of the same questionnaire, or standardized test, as data collection took place over a period of over 10 years, and new versions were developed over time. To investigate whether different versions were comparable in the models as fitted for the current study, different multigroup SEM analyses were conducted. More precisely, for MTMM model 1 and 2, three versions of the standardized tests for academic performance were used, for which measurement invariance was tested. For the structural equation model (SEM, i.e., serial mediation model), different versions were used for three variables: two versions for the ADHD symptom questionnaire, two versions for the standardized test of PS and WM (measured via the same test battery, and thus tested in one multigroup SEM), and again the three versions for the standardized tests for academic performance. For all these models, four levels of measurement invariance were tested (i.e., configural, metrical, scalar, and strict), but metrical invariance was sufficient for the current analyses, indicating factor loadings can be equalized across different versions. Additionally, for the structural models (i.e., serial mediation model), equality constraints on the direct and indirect pathways were imposed, to evaluate whether they could be equalized across test versions and thus direct and indirect pathways could be interpreted properly.

***Multitrait-multimethod (MTMM) models***

The model fit indices and the χ²-difference tests for the models investigating measurement invariance between the three versions of the standardized tests for academic performance can be found in Table S1 and S2. For both model 1 and 2, indices indicate excellent model fit all three versions of the standardized test of academic performance, and convergent validity was sufficient for all with factor loadings (range .613 to .871) larger than the method effects (range -.279 to .693) within the models of each test version. Moderate discriminant validity was found for the different academic subjects across all test versions (range correlations .554 to .811). Poor discriminant validity was found between reading and spelling across test versions (range correlations .898 to .962). Comparing model fit of the configural, metrical, scalar and strict model show data from the different versions of the standardized tests of academic performance could be collapsed into one for the final analyses. Only for model 2, model fit of the scale model was significantly worse compared to the metric model. Modification indices showed model fit would significantly improve when the equality constraint on the factor loadings of the two measures of reading performance would be freed. No significant modification indices were found related to the intercept equality constraint as added in the scalar model. Therefore, no modifications were made to the model.

**Table S1.** Comparison of configural, metric, scalar and strict models for standardized tests of academic performance in MTMM model 1.

| Model | χ² (df) | CFI | RMSEA | SMSR | Δχ² | Δdf | p-value |
| --- | --- | --- | --- | --- | --- | --- | --- |
| 1. Configural | 18.70 (15) | 0.996 | 0.039 | 0.024 |  |  |  |
| 2. Metric | 22.31 (23) | 1.000 | 0.000 | 0.031 | 3.61 | 8 | .891 |
| 3. Scalar | 32.62 (31) | 0.998 | 0.018 | 0.037 | 10.31 | 8 | .244 |
| 4. Strict | 45.88 (43) | 0.997 | 0.020 | 0.045 | 13.26 | 12 | .350 |

**p* < .05, ***p* < .01, ****p* < .001

**Table S2**. Comparison of configural, metric, scalar and strict models for standardized tests of academic performance in MTMM model 2.

| Model | χ² (df) | CFI | RMSEA | SMSR | Δχ² | Δdf | p-value |
| --- | --- | --- | --- | --- | --- | --- | --- |
| 1. Configural | 8.11 (9) | 1.000 | 0.000 | 0.022 |  |  |  |
| 2. Metric | 10.76 (15) | 1.000 | 0.000 | 0.041 | 2.65 | 6 | .851 |
| 3. Scalar | 23.84 (21) | 0.998 | 0.029 | 0.046 | 13.08 | 6 | .042* |
| 4. Strict | 28.11 (33) | 1.000 | 0.000 | 0.046 | 4.27 | 12 | .978 |

**p* < .05, ***p* < .01, ****p* < .001

***Serial mediation models***

The model fit indices and the χ²-difference tests for the different models investigating measurement invariance for the two versions of the ADHD symptom questionnaire, cognitive tests for PS and WM, and academic achievement, for all mediation models can be found in Table S3-S14. In the configural models, the structure of the serial mediation models was followed and showed excellent model fit for all three variables across all models (see Table S3-S14). For each model, the metric model, scalar model, and strict model showed excellent model fit, and none of the models resulted in significantly worse model fit compared to the previous model with one less constrain superimposed. Constraining the (in)direct effects to be equal did not lead to worse model fit either, for neither of the variables. Thus, these analyses show data from the different versions of ADHD symptom questionnaire, cognitive tests of PS and WM, and standardized tests for academic achievement could be collapsed into one for the final analyses. However, for serial mediation model 2 with inattention symptoms as predictor, model fit of the scalar model was significantly worse compared to the metric model. Similar to the measurement model as described above, modification indices showed model fit would improve when the equality constrain on the factor loadings of the two measures of reading performance would be freed. Thus, again no significant modification indices were found related to the intercept equality constraint as added in the scalar model. Therefore, no modifications were made to the model.

**Table S3**. Comparison of configural, metric, scalar and strict models for ADHD symptom questionnaire in serial mediation model 1 with inattention symptom severity as predictor, as well as the model imposing equality constraints on the (in)direct effects.

| Model | χ² (df) | CFI | RMSEA | SMSR | Δχ² | Δdf | p-value |
| --- | --- | --- | --- | --- | --- | --- | --- |
| 1. Configural | 77.82 (46) | .975 | .052 | .034 |  |  |  |
| 2. Metric | 82.17 (50) | .975 | .051 | .037 | 4.34 | 4 | .362 |
| 3. Scalar | 92.28 (56) | .972 | .051 | .039 | 10.12 | 6 | .120 |
| 4. Strict | 96.80 (64) | .975 | .053 | .040 | 4.51 | 8 | .808 |
| 5. (In)direct effects | 103.40 (75) | .978 | .039 | .042 | 6.60 | 11 | .831 |

**p* < .05, ***p* < .01, ****p* < .001

**Table S4**. Comparison of configural, metric, scalar and strict models for standardized tests of PS and WM in serial mediation model 1 with inattention symptom severity as predictor, as well as the model imposing equality constraints on the (in)direct effects.

| Model | χ² (df) | CFI | RMSEA | SMSR | Δχ² | Δdf | p-value |
| --- | --- | --- | --- | --- | --- | --- | --- |
| 1. Configural | 78.79 (46) | .975 | .053 | .033 |  |  |  |
| 2. Metric | 81.03 (50) | .976 | .050 | .033 | 2.25 | 4 | .690 |
| 3. Scalar | 89.52 (56) | .974 | .049 | .037 | 8.49 | 6 | .205 |
| 4. Strict | 89.34 (64) | .978 | .042 | .037 | 2.82 | 8 | .945 |
| 5. (In)direct effects | 101.17 (75) | .980 | .037 | .042 | 8.23 | 11 | .638 |

**p* < .05, ***p* < .01, ****p* < .001

**Table S5**. Comparison of configural, metric, scalar and strict models for standardized tests of academic achievement in serial mediation model 1 with inattention symptom severity as predictor, as well as the model imposing equality constraints on the (in)direct effects.

| Model | χ² (df) | CFI | RMSEA | SMSR | Δχ² | Δdf | p-value |
| --- | --- | --- | --- | --- | --- | --- | --- |
| 1. Configural | 101.10 (69) | .975 | .054 | .040 |  |  |  |
| 2. Metric | 107.68 (77) | .976 | .050 | .043 | 6.58 | 8 | .583 |
| 3. Scalar | 128.28 (89) | .969 | .052 | .049 | 20.60 | 12 | .057 |
| 4. Strict | 143.80 (105) | .970 | .048 | .050 | 15.52 | 16 | .487 |
| 5. (In)direct effects | 161.42 (127) | .973 | .041 | .058 | 17.62 | 22 | .729 |

**p* < .05, ***p* < .01, ****p* < .001

**Table S6**. Comparison of configural, metric, scalar and strict models for ADHD symptom questionnaire in serial mediation model 2 with inattention symptom severity as predictor, as well as the model imposing equality constraints on the (in)direct effects.

| Model | χ² (df) | CFI | RMSEA | SMSR | Δχ² | Δdf | p-value |
| --- | --- | --- | --- | --- | --- | --- | --- |
| 1. Configural | 47.25 (34) | .992 | .039 | .031 |  |  |  |
| 2. Metric | 48.24 (37) | .993 | .035 | .033 | 0.98 | 3 | .806 |
| 3. Scalar | 58.09 (42) | .990 | .039 | .035 | 9.85 | 5 | .080 |
| 4. Strict | 63.49 (50) | .991 | .033 | .037 | 5.41 | 8 | .713 |
| 5. (In)direct effects | 76.59 (65) | .993 | .027 | .041 | 13.10 | 15 | .595 |

**p* < .05, ***p* < .01, ****p* < .001

**Table S7**. Comparison of configural, metric, scalar and strict models for standardized tests of PS and WM in serial mediation model 2 with inattention symptom severity as predictor, as well as the model imposing equality constraints on the (in)direct effects.

| Model | χ² (df) | CFI | RMSEA | SMSR | Δχ² | Δdf | p-value |
| --- | --- | --- | --- | --- | --- | --- | --- |
| 1. Configural | 47.22 (34) | .992 | .039 | .031 |  |  |  |
| 2. Metric | 49.72 (37) | .992 | .037 | .032 | 2.50 | 3 | .476 |
| 3. Scalar | 57.91 (42) | .990 | .039 | .035 | 8.19 | 5 | .146 |
| 4. Strict | 59.72 (50) | .994 | .028 | .036 | 1.81 | 8 | .986 |
| 5. (In)direct effects | 76.34 (65) | .993 | .026 | .042 | 16.62 | 15 | .342 |

**p* < .05, ***p* < .01, ****p* < .001

**Table S8**. Comparison of configural, metric, scalar and strict models for standardized tests of academic achievement in serial mediation model 2 with inattention symptom severity as predictor, as well as the model imposing equality constraints on the (in)direct effects.

| Model | χ² (df) | CFI | RMSEA | SMSR | Δχ² | Δdf | p-value |
| --- | --- | --- | --- | --- | --- | --- | --- |
| 1. Configural | 65.50 (51) | .991 | .042 | .039 |  |  |  |
| 2. Metric | 71.79 (57) | .991 | .040 | .050 | 6.28 | 6 | .392 |
| 3. Scalar | 93.10 (67) | .983 | .049 | .056 | 21.31 | 10 | .019* |
| 4. Strict | 97.99 (83) | .990 | .033 | .054 | 4.89 | 16 | .996 |
| 5. (In)direct effects | 127.05 (113) | .991 | .028 | .058 | 29.06 | 30 | .514 |

**p* < .05, ***p* < .01, ****p* < .001

**Table S9**. Comparison of configural, metric, scalar and strict models for ADHD symptom questionnaire in serial mediation model 1 with hyperactivity/impulsivity symptom severity as predictor, as well as the model imposing equality constraints on the (in)direct effects.

| Model | χ² (df) | CFI | RMSEA | SMSR | Δχ² | Δdf | p-value |
| --- | --- | --- | --- | --- | --- | --- | --- |
| 1. Configural | 85.50 (46) | .970 | .058 | .035 |  |  |  |
| 2. Metric | 89.26 (50) | .970 | .056 | .037 | 3.76 | 4 | .440 |
| 3. Scalar | 95.04(56) | .970 | .053 | .038 | 5.78 | 6 | .448 |
| 4. Strict | 88.67 (64) | .973 | .046 | .039 | 3.80 | 8 | .874 |
| 5. (In)direct effects | 105.21 (75) | .977 | .040 | .043 | 6.36 | 11 | .848 |

**p* < .05, ***p* < .01, ****p* < .001

**Table S10**. Comparison of configural, metric, scalar and strict models for standardized tests of PS and WM in serial mediation model 1 with hyperactivity/impulsivity symptom severity as predictor, as well as the model imposing equality constraints on the (in)direct effects.

| Model | χ² (df) | CFI | RMSEA | SMSR | Δχ² | Δdf | p-value |
| --- | --- | --- | --- | --- | --- | --- | --- |
| 1. Configural | 90.53 (46) | .966 | .062 | .035 |  |  |  |
| 2. Metric | 92.55 (50) | .968 | .058 | .036 | 2.01 | 4 | .733 |
| 3. Scalar | 97.24 (56) | .969 | .054 | .037 | 4.69 | 6 | .584 |
| 4. Strict | 99.91 (64) | .973 | .047 | .038 | 2.68 | 8 | .953 |
| 5. (In)direct effects | 107.14 (75) | .976 | .041 | .043 | 7.23 | 11 | .780 |

**p* < .05, ***p* < .01, ****p* < .001

**Table S11**. Comparison of configural, metric, scalar and strict models for standardized tests of academic achievement in serial mediation model 1 with hyperactivity/impulsivity symptom severity as predictor, as well as the model imposing equality constraints on the (in)direct effects.

| Model | χ² (df) | CFI | RMSEA | SMSR | Δχ² | Δdf | p-value |
| --- | --- | --- | --- | --- | --- | --- | --- |
| 1. Configural | 114.29 (69) | .965 | .064 | .042 |  |  |  |
| 2. Metric | 120.16 (77) | .967 | .059 | .044 | 5.88 | 8 | .661 |
| 3. Scalar | 132.89 (89) | .966 | .055 | .047 | 12.72 | 12 | .390 |
| 4. Strict | 147.95 (105) | .967 | .050 | .050 | 15.06 | 16 | .520 |
| 5. (In)direct effects | 161.24 (127) | .973 | .041 | .056 | 13.29 | 22 | .925 |

**p* < .05, ***p* < .01, ****p* < .001

**Table S12**. Comparison of configural, metric, scalar and strict models for ADHD symptom questionnaire in serial mediation model 2 with hyperactivity/impulsivity symptom severity as predictor, as well as the model imposing equality constraints on the (in)direct effects.

| Model | χ² (df) | CFI | RMSEA | SMSR | Δχ² | Δdf | p-value |
| --- | --- | --- | --- | --- | --- | --- | --- |
| 1. Configural | 50.75 (34) | .989 | .044 | .032 |  |  |  |
| 2. Metric | 51.55 (37) | .991 | .039 | .033 | 0.80 | 3 | .850 |
| 3. Scalar | 57.35 (42) | .990 | .038 | .034 | 5.80 | 5 | .326 |
| 4. Strict | 62.93 (50) | .992 | .032 | .036 | 5.58 | 8 | .693 |
| 5. (In)direct effects | 76.07 (65) | .993 | .026 | .042 | 13.14 | 15 | .591 |

**p* < .05, ***p* < .01, ****p* < .001

**Table S13**. Comparison of configural, metric, scalar and strict models for standardized tests of PS and WM in serial mediation model 2 with hyperactivity/impulsivity symptom severity as predictor, as well as the model imposing equality constraints on the (in)direct effects.

| Model | χ² (df) | CFI | RMSEA | SMSR | Δχ² | Δdf | p-value |
| --- | --- | --- | --- | --- | --- | --- | --- |
| 1. Configural | 54.29 (34) | .987 | .049 | .032 |  |  |  |
| 2. Metric | 56.17 (37) | .988 | .045 | .033 | 1.87 | 3 | .599 |
| 3. Scalar | 60.83 (42) | .988 | .042 | .034 | 4.67 | 5 | .458 |
| 4. Strict | 62.74 (50) | .992 | .032 | .035 | 1.90 | 8 | .984 |
| 5. (In)direct effects | 77.94 (65) | .992 | .028 | .044 | 15.21 | 15 | .437 |

**p* < .05, ***p* < .01, ****p* < .001

**Table S14**. Comparison of configural, metric, scalar and strict models for standardized tests of academic achievement in serial mediation model 2 with hyperactivity/impulsivity symptom severity as predictor, as well as the model imposing equality constraints on the (in)direct effects.

| Model | χ² (df) | CFI | RMSEA | SMSR | Δχ² | Δdf | p-value |
| --- | --- | --- | --- | --- | --- | --- | --- |
| 1. Configural | 74.32 (51) | .985 | .053 | .040 |  |  |  |
| 2. Metric | 79.04 (57) | .986 | .049 | .047 | 4.72 | 6 | .581 |
| 3. Scalar | 93.17 (67) | .983 | .049 | .049 | 14.13 | 10 | .167 |
| 4. Strict | 98.04 (83) | .990 | .033 | .049 | 4.87 | 16 | .996 |
| 5. (In)direct effects | 121.53 (113) | .995 | .022 | .059 | 23.49 | 30 | .795 |

**p* < .05, ***p* < .01, ****p* < .001
