# Supplementary material for: Academic Achievement in Children with ADHD: the Role of Processing Speed and Working Memory
Source: Res Child Adolesc Psychopathol. 2025 Jul 15;53(10):1469–84. doi: 10.1007/s10802-025-01346-6 (PMC12521309; doi:10.1007/s10802-025-01346-6)
Supplement: Supplementary file 2 — Supplementary Material 2 [file 10802_2025_1346_MOESM2_ESM.docx]

**Title:** Academic achievement in children with ADHD: The role of processing speed and working memory

**Journal name:** Research on Child and Adolescent Psychopathology

**Author names:** An-Katrien Hulsbosch, Saskia Van der Oord* & Gail Tripp* (*joint last authors)

**Corresponding author:** An-Katrien Hulsbosch

**Supplement C: Serial mediation models for verbal and visual-spatial WM separately.**

**Table S15.** Fit indices, standardized beta coefficients for direct effects and unstandardized indirect effects with 95% bootstrap confidence intervals for the serial mediation models as shown in Figure 3, with verbal WM as indicator of WM.

|  | Inattention | | Hyperactivity/Impulsivity | |
| --- | --- | --- | --- | --- |
|  | Model 1 | Model 2 | Model 1 | Model 2 |
| **Model fit** |  |  |  |  |
| χ² (df) | 41.24 (23) | 25.173 (17) | 50.25 (23) | 30.48 (17) |
| CFI | 0.979 | 0.993 | 0.970 | 0.988 |
| RMSEA | 0.046 | 0.036 | 0.057 | 0.046 |
| SMSR | 0.031 | 0.027 | 0.032 | 0.028 |
| **Direct effects – standardized beta coefficient (S.E.)** | | |  |  |
| a | -.041 (.010) | -.041 (.010) | .054 (.009) | .054 (.009) |
| b_1_ | .128 (.067)* | .112 (.068)* | .129 (.066)* | .123 (.071) |
| b_2_ | .270 (.071)*** | .307 (.087)*** | .265 (.074)*** | .307 (.088)*** |
| b_3_ |  | .173 (.069)** |  | .173 (.070)** |
| c_1_ | -.149 (.011)** | -.134 (.011)* | .060 (.009) | .046 (.010) |
| c_2_ | -.147 (.040)* | -.139 (.015)* | .118 (.011) | .049 (.012) |
| c_3_ |  | -.187 (.013)** |  | .074 (.011) |
| d | -.062 (.010) | -.062 (.010) | -.003 (.008) | -.003 (.008) |
| e_1_ | .278 (.065)*** | .323 (.068)*** | .287 (.064)*** | .333 (.068)*** |
| e_2_ | .357 (.077)*** | .376 (.091)*** | .362 (.079)*** | .385 (.090)*** |
| e_3_ |  | .284 (.072)*** |  | .298 (.072)*** |
| f | .349 (.049)*** | .350 (.048)*** | .352 (.048)*** | .352 (.049)*** |
| g_1_ | .091 (.031) | .115 (.032)* | .097 (.034) | .117 (.036)* |
| g_2_ | -.178 (.040)** | -.166 (.044)** | -.151 (.043)* | -.162 (.047)* |
| g_3_ |  | .064 (.035) |  | .069 (.039) |
| *Single indirect pathways* [95% bootstrap confidence intervals] | | | | |
| a*b_1_ | -.001 [-.005, .002] | -.001 [-.005, .002] | .001 [-.001, .005] | .001 [-.001, .005] |
| a*b_2_ | -.003 [-.010, .004] | -.003 [-.012, .005] | .003 [-.003, .009] | .003 [-.003, .011] |
| a*b_3_ |  | -.001 [-.006, .002] |  | .002 [-.002, .006] |
| d*e_1_ | -.003 [-.010, .002] | -.004 [-.012, .003] | -.000 [-.005, .005] | -.000 [-.006, .006] |
| d*e_2_ | -.005 [-.014, .003] | -.005 [-.016, .004] | -.000 [-.007, .007] | -.000 [-.008, .007] |
| d*e_3_ |  | -.004 [-.011, .002] |  | -.000 [-.006, .005] |
| a*f | -.003 [-.010, .004] | -.003 [-.009, .004] | .003 [-.003, .010] | .003 [-.003, .010] |
| *Serial indirect pathways* [95% bootstrap confidence intervals] | | | | |
| a*f*e_1_ | -.001 [-.003, .001] | -.001 [-.004, .002] | .001 [-.001, .003] | .001 [-.001, .004] |
| a*f*e_2_ | -.001 [-.005, .002] | -.001 [-.005, .002] | .001 [-.001, .005] | .001 [-.001, .005] |
| a*f*e_3_ |  | -.001 [-.003, .001] |  | .001 [-.001, .004] |

**p* < .05, ***p* < .01, ****p* < .001
*Note.* For some CI or estimators, more decimals are displayed to indicate whether the estimate is different from zero or the CI interval contains the value zero.

**Table S16.** Fit indices, standardized beta coefficients for direct effects and unstandardized indirect effects with 95% bootstrap confidence intervals for the serial mediation models as shown in Figure 3, with spatial WM as indicator of WM.

|  | Inattention | | Hyperactivity/Impulsivity | |
| --- | --- | --- | --- | --- |
|  | Model 1 | Model 2 | Model 1 | Model 2 |
| **Model fit** |  |  |  |  |
| χ² (df) | 29.608 (23) | 21.613 (17) | 37.92 (23) | 27.154 (17) |
| CFI | 0.992 | 0.996 | 0.983 | 0.991 |
| RMSEA | 0.028 | 0.027 | 0.042 | 0.040 |
| SMSR | 0.027 | 0.024 | 0.028 | 0.026 |
| **Direct effects – standardized beta coefficient (S.E.)** | | |  |  |
| a | -.042 (.010) | -.041 (.010) | .055 (.009) | .054 (.009) |
| b_1_ | .162 (.064)** | .178 (.066)** | .169 (.063)** | .184 (.065)** |
| b_2_ | .346 (.072)*** | .391 (.085)*** | .345 (.072)*** | .396 (.085)*** |
| b_3_ |  | .236 (.067)*** |  | .242 (.065)*** |
| c_1_ | -.168 (.012)** | -.156 (.012)** | .055 (.009) | .044 (.010) |
| c_2_ | -.169 (.013)** | -.165 (.014)* | .123 (.011) | .052 (.012) |
| c_3_ |  | -.207 (.013)** |  | .075 (.011) |
| d | .008 (.009) | .008 (.009) | .039 (.007) | .038 (.007) |
| e_1_ | .216 (.062)*** | .196 (.065)** | .211 (.060)*** | .193 (.064)*** |
| e_2_ | .152 (.069)* | .161 (.079)* | .144 (.066)* | .157 (.080)* |
| e_3_ |  | .121 (.070) |  | .116 (.068) |
| f | .286 (.046)*** | .286 (.047)*** | .284 (.046)*** | .283 (.047)*** |
| g_1_ | .096 (.031) | .121 (.033)* | .099 (.034) | .120 (.036) |
| g_2_ | -.169 (.039)** | -.162 (.043)* | -.142 (.041)* | -.159 (.045)* |
| g_3_ |  | .069 (.035) |  | .073 (.038) |
| *Single indirect pathways* [95% bootstrap confidence intervals] | | | | |
| a*b_1_ | -.001 [-.006, .002] | -.001 [-.006, .002] | .002 [-.002, .006] | .002 [-.002, .006] |
| a*b_2_ | -.003 [-.011, .005] | -.004 [-.014, .005] | .003 [-.004, .011] | .004 [-.005, .013] |
| a*b_3_ |  | -.002 [-.008, .003] |  | .002 [-.002, .008] |
| d*e_1_ | .000 [-.004, .005] | .000 [-.004, .005] | .001 [-.002, .005] | .001 [-.002, .005] |
| d*e_2_ | .000 [-.003, .004] | .000 [-.004, .004] | .001 [-.001, .004] | .001 [-.001, .004] |
| d*e_3_ |  | .000 [-.003, .003] |  | .001 [-.001, .003] |
| a*f | -.002 [-.008, .003] | -.002 [-.008, .003] | .002 [-.003, .008] | .002 [-.003, .008] |
| *Serial indirect pathways* [95% bootstrap confidence intervals] | | | | |
| a*f*e_1_ | -.001 [-.002, .001] | -.000 [-.002, .001] | .001 [-.001, .002] | .000 [-.001, .002] |
| a*f*e_2_ | -.000 [-.002, .001] | -.000 [-.002, .001] | .000 [-.0005, .002] | .000 [-.001, .002] |
| a*f*e_3_ |  | -.000 [-.001, .0004] |  | .000 [-.0003, .001] |

**p* < .05, ***p* < .01, ****p* < .001
*Note.* For some CI or estimators, more decimals are displayed to indicate whether the estimate is different from zero or the CI interval contains the value zero.
