# Supplementary material for: Academic Achievement in Children with ADHD: the Role of Processing Speed and Working Memory
Source: Res Child Adolesc Psychopathol. 2025 Jul 15;53(10):1469–84. doi: 10.1007/s10802-025-01346-6 (PMC12521309; doi:10.1007/s10802-025-01346-6)
Supplement: Supplementary file 3 — Supplementary Material 3 [file 10802_2025_1346_MOESM3_ESM.docx]

**Title:** Academic achievement in children with ADHD: The role of processing speed and working memory

**Journal name:** Research on Child and Adolescent Psychopathology

**Author names:** An-Katrien Hulsbosch, Saskia Van der Oord* & Gail Tripp* (*joint last authors)

**Corresponding author:** An-Katrien Hulsbosch

**Supplement C: Correlation matrix of included variables.**

**Table S17.** Correlation matrix of included variables.

|  | Age | Inattentive symptoms | Hyperactivity/impulsivity symptoms | Processing speed index | Working memory index | ST – Reading | ST – Math | ST – Spelling | PR – Reading | PR – Math | TR – Reading | TR – Math | TR – Spelling |
| --- | --- | --- | --- | --- | --- | --- | --- | --- | --- | --- | --- | --- | --- |
| Age | 1 |  |  |  |  |  |  |  |  |  |  |  |  |
| Inattentive symptom | **.122 **** | 1 |  |  |  |  |  |  |  |  |  |  |  |
| Hyperactivity/ impulsivity symptom | **-.266 ***** | **.351 ***** | 1 |  |  |  |  |  |  |  |  |  |  |
| Processing speed index | -.036 | -.086 | .073 | 1 |  |  |  |  |  |  |  |  |  |
| Working memory index | -.001 | -.046 | .065 | **.369 ***** | 1 |  |  |  |  |  |  |  |  |
| ST – Reading | .025 | **-.098 *** | .017 | **.131 **** | **.260 ***** | 1 |  |  |  |  |  |  |  |
| ST – Math | **-.146 **** | **-.126 **** | .056 | **.297 ***** | **.344 ***** | **.319 ***** | 1 |  |  |  |  |  |  |
| ST – Spelling | .018 | **-.122 **** | .044 | **.165 ***** | **.257 ***** | **.774 ***** | **.391 ***** | 1 |  |  |  |  |  |
| PR – Reading | -.021 | **-.149 ***** | .037 | **.100 *** | **.257 ***** | **.565 ***** | **.292 ***** | **.470 ***** | 1 |  |  |  |  |
| PR – Math | **-.145 **** | **-.143 **** | **.197 ***** | **.222 ***** | **.249 ***** | **.255 ***** | **.474 ***** | **.212 ***** | **.383 ***** | 1 |  |  |  |
| TR – Reading | .068 | -.082 | **.099 *** | **.179 ***** | **.285 ***** | **.623 ***** | **.336 ***** | **.545 ***** | **.683 ***** | **.350 ***** | 1 |  |  |
| TR – Math | -.074 | -.071 | **.185 ***** | **.239 ***** | **.288 ***** | **.326 ***** | **.463 ***** | **.318 ***** | **.407 ***** | **.519 ***** | **.554 ***** | 1 |  |
| TR – Spelling | -.015 | **-.162 ***** | **.119 *** | **.207 ***** | **.203 ***** | **.538 ***** | **.311 ***** | **.541 ***** | **.534 ***** | **.286 ***** | **.688 ***** | **.477 ***** | 1 |

*Note.* ST = standardized test; PR = parent rating; TR = teacher rating.

**p* < .05, ***p* < .01, ****p* < .001
