# Supplementary material for: Academic Achievement in Children with ADHD: the Role of Processing Speed and Working Memory
Source: Res Child Adolesc Psychopathol. 2025 Jul 15;53(10):1469–84. doi: 10.1007/s10802-025-01346-6 (PMC12521309; doi:10.1007/s10802-025-01346-6)
Supplement: Supplementary file 4 — Supplementary Material 4 [file 10802_2025_1346_MOESM4_ESM.docx]

**Title:** Academic achievement in children with ADHD: The role of processing speed and working memory

**Journal name:** Research on Child and Adolescent Psychopathology

**Author names:** An-Katrien Hulsbosch, Saskia Van der Oord* & Gail Tripp* (*joint last authors)

**Corresponding author:** An-Katrien Hulsbosch

**Supplement D: Reversed serial mediation models with WM as the first, and PS as the second mediator in the serial mediation.**

**Table S18.** Fit indices, standardized beta coefficients for direct effects and unstandardized indirect effects with 95% bootstrap confidence intervals for the serial mediation models with WM as the first, and PS as the second mediator.

|  | Inattention | | Hyperactivity/Impulsivity | |
| --- | --- | --- | --- | --- |
|  | Model 1 | Model 2 | Model 1 | Model 2 |
| **Model fit** |  |  |  |  |
| χ² (df) | 34.54 (23) | 28.178 (17) | 46.42 (23) | 30.48 (17) |
| CFI | 0.991 | 0.993 | 0.982 | 0.988 |
| RMSEA | 0.032 | 0.036 | 0.045 | 0.046 |
| SMSR | 0.024 | 0.025 | 0.027 | 0.028 |
| **Direct effects – standardized beta coefficient (S.E.)** | | |  |  |
| a | -.047 (.008) | -.046 (.008) | .066 (.006) | .066 (.006) |
| b_1_ | .312 (.054)*** | .302 (.056)*** | .311 (.053)*** | .302 (.057)*** |
| b_2_ | .332 (.061)*** | .364 (.072)*** | .325 (.062)*** | .360 (.073)*** |
| b_3_ |  | .257 (.057)*** |  | .258 (.058)*** |
| c_1_ | -.134 (.010)** | -.111 (.010)* | .065 (.008) | .047 (.008) |
| c_2_ | -.114 (.011)* | -.083 (.012) | .138 (.009)* | .079 (.010) |
| c_3_ |  | -.172 (.011)** |  | .071 (.009) |
| d | -.069 (.008) | -.068 (.008) | .049 (.007) | .049 (.007) |
| e_1_ | .068 (.055) | .086 (.056) | .077 (.053) | .093 (.056) |
| e_2_ | .228 (.061)*** | .247 (.067)*** | .228 (.061)*** | .250 (.068)*** |
| e_3_ |  | .150 (.059)** |  | .159 (.057)** |
| f | .366 (.041)*** | .366 (.041)*** | .366 (.042)*** | .366 (.041)*** |
| g_1_ | .050 (.029) | .050 (.029) | .053 (.030) | .050 (.031) |
| g_2_ | -.155 (.034)** | -.139 (.036)* | -.130 (.036)* | -.128 (.037)* |
| g_3_ |  | .037 (.032) |  | .036 (.034) |
| *Single indirect pathways* [95% bootstrap confidence intervals] | | | | |
| a*b_1_ | -.003 [-.009, .003] | -.003 [-.008, .002] | .003 [-.001, .008] | .003 [-.001, .008] |
| a*b_2_ | -.003 [-.010, .003] | -.004 [-.012, .003] | .004 [-.001, .009] | .004 [-.001, .011] |
| a*b_3_ |  | -.002 [-.008, .002] |  | .003 [-.001, .007] |
| d*e_1_ | -.001 [-.003, .001] | -.001 [-.004, .0004] | .001 [-.0005, .003] | .001 [-.001, .003] |
| d*e_2_ | -.003 [-.008, .0004] | -.004 [-.009, .001] | .002 [-.002, .006] | .002 [-.002, .007] |
| d*e_3_ |  | -.002 [-.006, .0003] |  | .001 [-.001, .004] |
| a*f | -.003 [-.010, .003] | -.003 [-.010, .003] | .004 [-.001, .009] | .004 [-.001, .008] |
| *Serial indirect pathways* [95% bootstrap confidence intervals] | | | | |
| a*f*e_1_ | -.000 [-.001, .001] | -.000 [-.001, .0003] | .000 [-.0001, .001] | .000 [-.0001, .001] |
| a*f*e_2_ | -.001 [-.003, .0003] | -.001 [-.003, .001] | .001 [-.0002, .003] | .001 [-.0002, .003] |
| a*f*e_3_ |  | -.001 [-.002, .0004] |  | .001 [-.0001, .002] |

**p* < .05, ***p* < .01, ****p* < .001
*Note.* For some CI or estimators, more decimals are displayed to indicate whether the estimate is different from zero or the CI interval contains the value zero.
